# Aquatic plant Azolla as the universal feedstock for biofuel production

**DOI:** 10.1186/s13068-016-0628-5

**Published:** 2016-10-18

**Authors:** Ana F. Miranda, Bijoy Biswas, Narasimhan Ramkumar, Rawel Singh, Jitendra Kumar, Anton James, Felicity Roddick, Banwari Lal, Sanjukta Subudhi, Thallada Bhaskar, Aidyn Mouradov

**Affiliations:** 1 School of Sciences, RMIT University, Bundoora, VIC Australia; 2 Thermo-Catalytic Processes Area (TPA), Bio-Fuels Division (BFD), CSIR-Indian Institute of Petroleum, Dehradun, Uttarakhand 248005 India; 3 The Energy and Resources Institute, New Delhi, 110 003 India; 4 School of Architecture and Design, RMIT University, Melbourne, Australia; 5 School of Engineering, RMIT University, Melbourne, Australia

**Keywords:** Azolla, Biofuel, Bio-ethanol, Bio-hydrogen, Bioremediation, Feedstock, Hydrothermal liquefaction, Wastewater treatment

## Abstract

**Background:**

The quest for sustainable production of renewable and cheap biofuels has triggered an intensive search for domestication of the next generation of bioenergy crops. Aquatic plants which can rapidly colonize wetlands are attracting attention because of their ability to grow in wastewaters and produce large amounts of biomass. Representatives of Azolla species are some of the fastest growing plants, producing substantial biomass when growing in contaminated water and natural ecosystems. Together with their evolutional symbiont, the cyanobacterium *Anabaena azollae,* Azolla biomass has a unique chemical composition accumulating in each leaf including three major types of bioenergy molecules: cellulose/hemicellulose, starch and lipids, resembling combinations of terrestrial bioenergy crops and microalgae.

**Results:**

The growth of *Azolla filiculoides* in synthetic wastewater led up to 25, 69, 24 and 40 % reduction of NH_4_–N, NO_3_–N, PO_4_–P and selenium, respectively, after 5 days of treatment. This led to a 2.6-fold reduction in toxicity of the treated wastewater to shrimps, common inhabitants of wetlands. Two Azolla species, *Azolla filiculoides* and *Azolla pinnata,* were used as feedstock for the production of a range of functional hydrocarbons through hydrothermal liquefaction, bio-hydrogen and bio-ethanol. Given the high annual productivity of Azolla, hydrothermal liquefaction can lead to the theoretical production of 20.2 t/ha-year of bio-oil and 48 t/ha-year of bio-char. The ethanol production from *Azolla filiculoides,* 11.7 × 10^3^ L/ha-year, is close to that from corn stover (13.3 × 10^3^ L/ha-year), but higher than from miscanthus (2.3 × 10^3^ L/ha-year) and woody plants, such as willow (0.3 × 10^3^ L/ha-year) and poplar (1.3 × 10^3^ L/ha-year). With a high C/N ratio, fermentation of Azolla biomass generates 2.2 mol/mol glucose/xylose of hydrogen, making this species a competitive feedstock for hydrogen production compared with other bioenergy crops.

**Conclusions:**

The high productivity, the ability to grow on wastewaters and unique chemical composition make Azolla species the most attractive, sustainable and universal feedstock for low cost, low energy demanding, near zero maintenance system for the production of a wide spectrum of renewable biofuels.

**Electronic supplementary material:**

The online version of this article (doi:10.1186/s13068-016-0628-5) contains supplementary material, which is available to authorized users.

## Background

The deleterious consequences of the extensive usage of arable lands to produce biofuels have triggered an intensive search for the next generation of energy crops which can grow on marginal lands not used for food production, forestry, or other uses of social value, including nature conservation. The use of wastewater as a source of reclaimed water and key nutrients for growing energy crops would significantly reduce the cost and energy requirement for biofuel production. Since the utilization of wastewater is very limited for most of terrestrial crops, attention has been shifted towards the use of aquatic plants and microalgae. Microalgae have shown obvious advantages in the production of biofuels compared with energy crops. Apart from their high growth rates and substantial lipid/triacylglycerol (TAG) yields, microalgae can grow in wastewater (animal, municipal and mining wastewaters) efficiently removing the primary nutrients (C, N and P), heavy metals and micropollutants, and they do not compete with crops for arable lands [1–3]. However, one of the major challenges of microalgal biotechnology for biofuel production is the high cost of harvesting [1, 4].

Recently, aquatic plants which rapidly colonize lakes and contaminated wetlands have attracted significant attention because of their high growth rates, high biomass production, bioremediation capacity, easy maintenance and easy harvest [5–7]. Growing these plants in wastewater can significantly improve the water quality by accumulating nutrients and heavy metals, and by regulating the oxygen balance [8, 9]. Various aquatic floating plants have been proposed as agents of choice for the bioremediation of wastewaters because of these important features. Among them, the most used are representatives of the *Lemnaceae* or duckweed which have been employed for over 20 years to recover nutrients from wastewaters and conversion of the generated biomass into biofuels [10–17]. The average annual yield of duckweed is 39.2–44 t dw/ha-year which is higher than the yields of the main bioenergy grasses: switchgrass (5.2–26 t/ha-year), poplar (9–15 t/ha-year) and miscanthus (5.0–44 t/ha-year) (Additional file 1: Table S1). Apart from efficient rates of nitrogen (N) and phosphorus (P) uptake, duckweed species can accumulate microelements and heavy metals to concentrations 100,000 times greater than in the surrounding water [18]. Because of their high growth rates and accumulation of starch (up to 45.7 % DW), duckweed species were used as feedstocks for bio-ethanol production [19, 20].

Azolla (mosquito fern, water fern) is a genus with seven species found in ponds, ditches, and wetlands throughout the world, from temperate to tropical regions [21] (Additional file 2: Figure S1). This aquatic plant is one of the fastest growing plants capable of doubling its biomass every 5–6 days [21]. Growing on artificial media, wastewaters and maturation ponds, its productivity can vary between 2.9 and 5.8 g dw/m^2^-day (10.5–21.1 t dw/ha-year, Additional file 1: Table S1) [22–25]. Growing in natural ecosystems, rivers, lagoons and irrigation channels, Azolla plants can bloom with a rate up to 25.6–27.4 g dw/m^2^-day (93.4–100 t dw/ha-year) [25]. Their growth in wastewaters is associated with the removal of the key wastewater nutrients such as N and P, with rates of up to 2.6 t N/ha-year and 0.434 t P/ha-year, respectively [23–26]. Azolla can also grow efficiently in nitrogen-depleted media using the nitrogen fixing capacity of its symbiont, the endophytic cyanobacterium, *Anabaena azollae* Strasburger (*A. azollae*), which grows within its leaf cavities (Additional file 2: Figure S1). As a result, Azolla’s growth is associated with the fixation of nitrogen of up to 1.1 t/ha-year, which is significantly higher than the nitrogen fixation rate of legumes (0.4 t N/ha-year) [21, 25, 27].

The chemical composition of Azolla comprises a unique mixture of key molecules found in lignocellulosic, starch- and oil-producing terrestrial bioenergy crops and microalgal/cyanobacterial representatives. *A. filiculoides* contains starch (up to 6 % dw), cellulose/hemicellulose (up to 35 % dw) and lipids (8 % dw) (Additional file 1: Table S1, Additional file 3: Table S2). As a result, growing under natural conditions (with biomass production up to 100 t dw/ha-year) Azolla biomass can accumulate up to 6 t dw/ha-year of starch and 34 t dw/ha-year of cellulose/hemicellulose. The Azolla biomass can also accumulate up to 8 t/ha-year of neutral lipids which is higher than from soybean, sunflower, rapeseed and oil palm [28–30]. Furthermore, the composition of the fatty acid methyl esters produced after transesterification of Azolla/*A. azollae* lipids, C16:0, C18:2 and C18:3, meets the crucial requirements of fuel density, cetane number and iodine value for biodiesel set by the EN14214 standard [28].

The unique chemical composition makes Azolla species an attractive feedstock for a range of biofuels. We have previously shown that pyrolysis of *A. filiculoides* grown in wastewater produces up to 33 % of bio-oil containing a range of petrochemicals, including straight-chain C10–C21 alkanes which can be directly used as a glycerine-free component of biodiesel [24]. In this work, we used *A. filiculoides* for the treatment of a synthetic wastewater which simulates the characteristics of effluents from typical textile dyeing, finishing and laundry detergent production industries. To increase the toxicity to common water inhabitants, such as crustaceans (*P. australiensis*), this wastewater was supplemented with selenium (SeO_2_, 0.8 mg/L). For the first time, the *A. filiculoides* biomass was used as feedstock for production of a range of functional hydrocarbons through hydrothermal liquefaction (HTL), and for bio-hydrogen production. Two of the most common Azolla species, *A. filiculoides,* and *A. pinnata*, found in temperate, warm and tropical regions and common for Australia and India, were used as feedstock for production of bio-ethanol. Our results showed that the ability of Azolla species to treat wastewater, and their unique chemical compositions resembling combinations of terrestrial bioenergy crops and microalgae/cyanobacteria, makes this plant the most attractive universal feedstock for a low cost, low energy demanding, near zero maintenance system for the production of a wide spectrum of biofuels.

## Results and discussion

### Treatment of synthetic wastewater with *Azolla filiculoides*

**Fig. 1:**
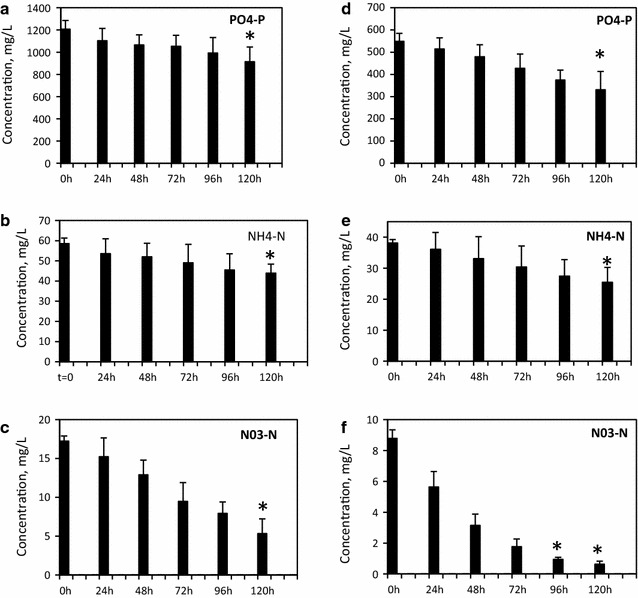
Reductions of concentrations of PO_4_–P, NH_4_−N and NO_3_–N in 100 % (**a**, **b**, **c**, respectively) and 50 % (**d**, **e**, **f**, respectively) SeSW by *A. filiculoides.* Significance levels: *p < 0.05

For the bioremediation experiment, selenium-rich synthetic wastewater (SeSW) was prepared by mixing a high concentration of phosphates (PO_4_–P), 1.3 g/L with a moderate amount ammonia (NH_4_–N), 55 mg/L and low concentration of nitrates (NO_3_–N), 15 mg/L (Additional file 4: Table S3). This composition simulates the characteristics of effluents from typical textile dyeing, finishing and laundry detergent production industries [31, 32]. The wastewater was supplemented with 0.8 mg/L of SeO_2_. In this experiment, we used 5-day treatment of SeSW by *A. filiculoides*. Growth rates of some of aquatic plants growing on wastewaters normally increase exponentially after a lag phase observed over the first 4–5 days when the biomass does not change significantly [20]. This period, however, is associated with the intensive absorption of the key nutrients from wastewaters, which leads to the strong exponential growth of aquatic plants after the lag phase [33, 34]. Removal of nutrients in the SeSW over the first 5 days of treatment by *A. filiculoides* did not lead to statistically significant changes (*P* ≤ 0.05) in biomass production (0.3 ± 0.1 g dw). However, it led to up to 25.4 % uptake of NH_4_–N, 69.5 % uptake of NO_3_–N and 24.3 % uptake of PO_4_–P from 100 % SeSW (Table 1; Fig. 1). Diluted (50 %) SeSW was less stressful to the *A. filiculoides* as reflected in the higher rates of nutrient uptake: 33.4, 93 and 39.8 % for NH_4_–N, NO_3_–N and PO_4_–P, respectively. Absorption rates of nutrients by *A. filiculoides* were reported earlier by several research groups [21, 23–26, 35, 36].

**Table 1:** Nutrients removal from SeSW by *Azolla filiculoides*

SeSW	Final biomass, gDW	NH_4_ uptake				PO_4_ uptake				NO_3_ uptake			
		NH4, mg/L, final	NH4 uptake, %	NH4 uptake rate, mg/L-day	NH4 uptake, mg/g DW-day	PO4-, mg/L, final	PO4-P uptake, %	PO4-P uptake rate, mg/L- day	PO4- uptake, mg/gDW-day	NO3-, mg/L, final	NO3-uptake, %	NO3-uptake rate, mg/L-day	NO3- uptake, mg/gDW-day
100 %	0.3 ± 0.01	43.9 ± 7.9	25.4 ± 8.9	2.96 ± 0.5	9.8 ± 2.2	914.21 ± 65.9	24.3 ± 4.3	58.4 ± 8.2	194 ± 42.9*	5.2 ± 0.3	69.5 ± 10.8	2.4 ± 0.1	7.9 ± 2.5*
50 %	0.3 ± 0.01	25.4 ± 5.5	33.4 ± 12	2.6 ± 0.9	8.4 ± 3.9	330.9 ± 23.0	39.8 ± 5.4	43.6 ± 7.9	145.1 ± 11*	0.6 ± 0.2	93.1 ± 9.8	1.62 ± 0.08	5.37 ± 1.1*

* Significance levels: p < 0.05

**Fig. 2:**
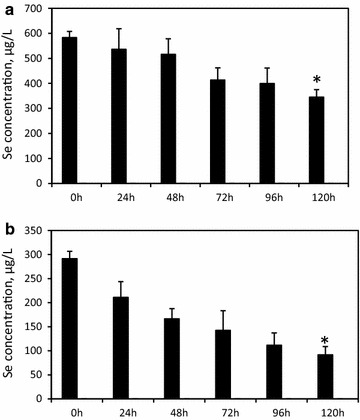
Reduction in concentration of selenium in 100 % (**a**) and 50 % (**b**) SeSW treated by *A. filiculoides*. Significance levels: *p < 0.05

Treatment of SeSW with *A. filiculoides* led to 40 % uptake of Se from 100 % SeSW and 76 % uptake from 50 % SeSW with absorption rates of 47.6 µg Se/L-day and 39.9 µg Se/L-day, respectively (Fig. 2; Table 2). This was correlated with accumulation rates of 158.8 µg of Se/g dw-day in *A. filiculoides* from 100 % SeSW and 133.5 µg of Se/g dw-day from 50 % SeSW (Table 2). This absorption efficiency represents 85 and 83 % of the theoretical maximum absorption value for 50 % SeSW and 100 % SeSW, respectively. An approximately similar absorption rate of Se was shown by another Azolla representative, *Azolla caroliniana,* which absorbed up to 1 mg Se/g dw from 2.5 mg/L Se solution over 2 weeks of treatment [37]. This is higher than for other aquatic plants, *Salvinia rotundifolia* (0.7 mg Se/g dw), *Lemna minor* (500 mg Se/g dw) and *Eichhornia* (300 mg Se/g dw) [37].

**Table 2:** Se absorption by *Azolla filiculoides* biomass

SeSW	Se concentration in biomass, mg/gDW	Se absorbtion by 0.3 g of biomass, µg	Theoretical absorption, µg^a^	Se absorption, %	Absorption rate, Se/gDW-day
Control	0.072 ± 0.03	21.6 ± 3.01	NA	NA	NA
50 %	0.358 ± 0.12*	85.8 ± 18.0^b^	101	85 %	133.5 ± 19.4*
100 %	0.404 ± 0.18*	99.4 ± 17.4^b^	119	83 %	158.8 ± 27.1*

Control, Se accumulation in Azolla biomass at time 0

*NA* not analysed

Significance levels: p < 0.05

^a^Theoretical absorption by 0.3 g DW of *A. filiculoides* calculated based on reduction of Se amount in 500 ml of wastewater after 5 days of treatment

^b^net amount of Se accumulated in 0.3 g of dry biomass after 5 days growth in SeSW (with 21.6 µg of Se subtracted)

The survival rate of *P. australiensis* exposed to dilutions of SeSW is shown in Additional file 5: Figure S2. There was zero survival (0 %) when they were exposed for 96 h to 100 % untreated (control) SeSW. No live shrimps were also observed in either 80 % or 50 % SeSW. After treatment with *A*. *filiculoides*, the toxicity of the SeSW was reduced 2.6-fold with LC_50_ increased from 11.22 to 29.80 %. This indicated that when 100 % SeSW was treated by *A. filiculoides,* its toxicity was significantly reduced after only 5 days of treatment.

### Biofuel production from Azolla

#### Ultimate and proximate analyses of *Azolla filiculoides*

The results of the proximate and ultimate analyses of the *A. filiculoides* sample, including the total content of volatiles, moisture, fixed carbon and ash (i.e. inorganic components of the samples), are summarized in Additional file 6: Table S4. Total volatiles collected at 950 °C represent 88 % of the total product. The total product had 11 % moisture, 7.3 % ash and 4.4 % fixed carbon. Proximate analysis showed the key elements to comprise 46.2 % C, 7.4 % H, 3.0 % N, 43.2 % O and 0.2 % S (calculated by difference).

#### Thermogravimetric analyses of *Azolla filiculoides*

**Fig. 3:**
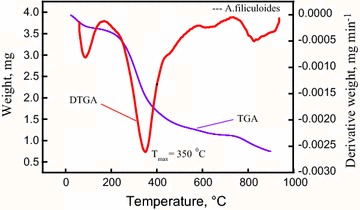
Thermogravimetric analyses of *A. filiculoides* biomass

TGA-DTG analysis of the *A. filiculoides* sample was carried out at a temperature range of 23–900 °C with a heating rate of 10 °C/min under a nitrogen atmosphere and is shown in Fig. 3. TGA and DTG curves revealed the three stages of decomposition of the *A. filiculoides* in the pyrolysis process; similar results were reported by Agrawal and Chakraborty [38]. The biomass underwent three phases of weight loss, one between 154 and 160 °C, the second at 180 and 580 °C and the third at around 580 and 900 °C. The first zone represents a slight weight (7 %) loss caused by dehydration of the biomass sample. Most of the weight loss (51 %) due to pyrolysis took place in the second zone, where most of the volatiles are released; the strong peak denotes the decomposition of proteins, carbohydrates, and lipids [39]. In the third zone, there is decomposition of the carbonaceous product, and thus the weight loss is mainly due to gasification where highly non-volatile carbon compounds vaporize forming CO and CO_2_ due to the high temperatures [38].

#### Hydrothermal liquefaction of *Azolla filiculoides*

**Fig. 4:**
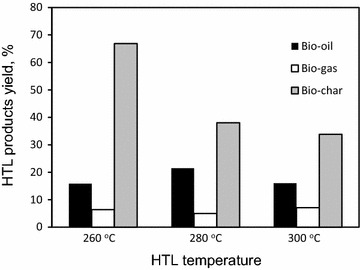
Product distribution from hydrothermal liquefaction of *A. filiculoides* biomass. Conversion efficiencies: 260 °C: (33.17 %; other yield 10.92 %); 280 °C: (62.0 %; other yield 35.5 %); 260 °C: (66.17 %; other yield, 43.0 %)

Hydrothermal liquefaction of *A. filiculoides* was performed using distilled water as solvent at 260, 280 and 300 °C with residence times of 15 min. Reaction conditions were selected based on our earlier studies and literature to understand the effect of temperature on the bio-oil product yield of *A. filiculoides* under subcritical water [40]. The product distribution of the HTL-treated *A. filiculoides* sample is presented in Fig. 4. The total bio-oil yields were 15.83, 21.50 and 16.0 % at 260, 280 and 300 °C, respectively; thus the maximum yield was obtained at 280 °C. The total bio-oil was composed of the ether fraction (Bio-oil1) obtained from extraction of the liquid portion, and the acetone fraction (Bio-oil2) obtained from extraction of the solid fraction (“Methods” section). Bio-oil2 had high viscosity and was seen to be a tarry liquid. The solid residue yields decreased continuously from 66.83 to 33.83 % as the temperature increased from 260 to 300 °C. The yield of the gases decreased in the transition from 260 to 280 °C. When the temperature was higher than 280 °C, the yield of gases increased to 7.1 % [40]. HTL of *A. filiculoides* biomass showed a similar distribution of the two major products, biogas and bio-char, with data obtained after pyrolysis [41] with an average biogas of 6.05 and 12 % for HTL and pyrolysis, respectively, and bio-char around 50 % for both. HTL generated a higher level of bio-oil, up to 21.5 % (14 % for pyrolysis). Similar results were obtained when compared with the yields of bio-oil obtained from both pyrolysis and HTL of microalgae [41].

In addition to the material balance data, conversion on the basis of total organic carbon (TOC) obtained after hydrothermal liquefaction at different temperatures was investigated, and the results are presented in Additional file 7: Table S5. There was 46.9 % organic carbon in the feed which translates to 2.8 g of organic carbon in the 6 g of the sample used for hydrothermal liquefaction. The conversion regarding organic carbon increased from 13.93 to 48.93 % with increase in temperature from 260 to 300 °C. The results indicated that at a lower temperature, the decomposition of biomass was incomplete and left a large amount of unreacted biomass which may suppress the bio-oil formation. The rise in temperature was able to accelerate the decomposition of the feedstock and benefited the bio-oil formation; however, a further increase in temperature, higher than 280 °C in our experiments, would break down the previously formed bio-oil/intermediates to gases and water-soluble products and thus led to a decrease in the bio-oil yield. The reducing yield of the solid residue (bio-char) suggested an increase in the overall biomass conversion when the temperature was raised from 260 to 300 °C.

### Fourier transform–infrared spectroscopy of bio-oil

**Fig. 5:**
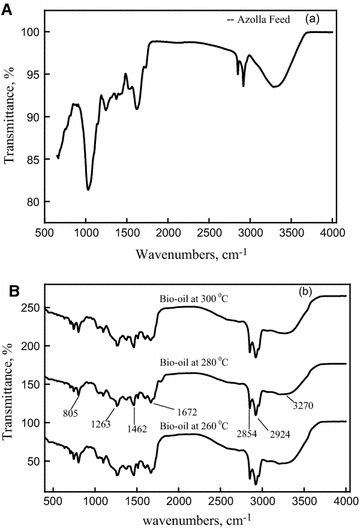
Fourier transform–infrared spectroscopy (FT-IR) of **A** *A. filiculoides* feed and **B** bio-oil (bio-oil1) from hydrothermal liquefaction of *A. filiculoides* at 260, 280 and 300 °C temperatures

FT-IR spectra of the *A. filiculoides* feed and bio-oil1 obtained from HTL at 260, 280 and 300 °C are shown in Figs. 5A and B. The broad band at around 3200–3405 cm^−1^ is attributed to the O–H or N–H stretching vibration caused by water or O–H groups or N–H groups present in bio-oil [42] (Fig. 5B). A broad absorbance was displayed at around 3314 cm^−1^ for the raw material, which indicated a high content of carbohydrates and proteins [43] (Fig. 5A).

The bio-oils showed a weaker absorbance at 3200–3405 cm^−1^, suggesting that both carbohydrates and proteins were decomposed in the HTL process (Fig. 5B). The bands from 2854 to 2950 cm^−1^ were stronger for all the bio-oils due to the C–H stretching vibrations, indicating the presence of alkyl C–H groups. The C=O stretching vibration around at 1645–1720 cm^−1^ in the bio-oils indicates the presence of ketones, aldehydes, esters or acids [42]. The bending vibration bands at around 1580–1650 cm^−1^ indicate the presence of the N–H groups of amine. The bands in the region from 1430 to 1480 cm^−1^ were attributed to α-CH_2_ bending vibrations present in the bio-oils. The presence of C–N stretching bands around at 1266–1342 cm^−1^ in the bio-oils is due to aromatic amine groups. Also, some other absorbance peaks appearing at 780–850 cm^−1^ are ascribed to the C–H out of plane bending vibrations from aromatics [42]. The band at 1040 cm^−1^ appeared only in the absorption profile of the *A. filiculoides* feed, and could be C–O connected with hydroxyl groups which were dehydrated after liquefaction. Overall, the spectra of the bio-oil1 samples from HTL at the different temperatures show the same peaks, indicating the presence of the same functional groups, and were the various peaks obtained in the NMR spectra.

### NMR analysis of bio-oil

**Fig. 6:**
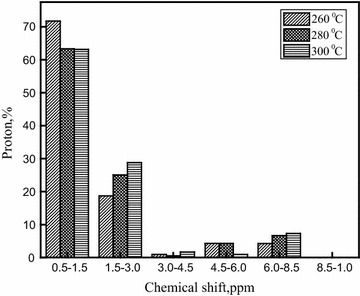
^1^H NMR spectral distribution of functional groups present in ether-soluble bio-oil (bio-oil1) from hydrothermal liquefaction of *A. filiculoides* at 260, 280 and 300 °C temperatures

NMR analysis of the bio-oil1 samples was undertaken to understand the ratios of chemical environments of the protons. The NMR spectra provided complementary functional group information to the FTIR spectra and the ability to quantify and compare integration areas between spectra. Similar to FT-IR, the ^1^H NMR spectra showed a high percentage of aliphatic functional groups for all bio-oils, and a summary of integrated peak area regions assigned to different functional group classes is provided in Fig. 6. The most up field region of the spectra, from 0.5 to 1.5 ppm, represents aliphatic protons attached to carbon atoms at least two bonds away from a C=C or heteroatom (O or N). The next integral region from 1.5 to 3.0 ppm represents protons on aliphatic carbon atoms that may be bonded to a C=C double bond. All the bio-oils had a higher percentage of protons in the spectral region from 0.5 to 3.0 ppm. They had a higher percentage (63.15–71.76 %) of protons in the region from 0.5 to 1.5 than the region from 1.5 to 3.0 (18.73–28.8 %) which is possibly due to the large number of nitrogenous and oxygenated compounds that have been shown to resonate in this area [42, 44] which may be derived from the high protein content of the feedstock.

The next portion of the ^1^H NMR spectrum at 3.0–4.5 ppm represents methoxyl protons [45] or a methylene group that joins two aromatic rings; very low proton percentages were observed in this region. All bio-oils displayed a low percentage of methoxy/carbohydrate functionality (4.5–6.0 ppm). In this region, the maximum proton percentage values of the bio-oils were 4.33 and 4.32 % for liquefactions at 260 and 280 °C, the markedly lower value of 0.94 % for liquefaction at 300 °C. The region of the spectrum between 6.0 and 8.5 ppm corresponds to the aromatic region. The maximum proton content of around 7.2 % in this region was obtained for the bio-oil made at 300 °C. Aromatic/heteroaromatic functionality was observed in all the bio-oils (6.0–8.5 ppm) in agreement with the findings from FT-IR spectroscopy. The downfield spectrum regions (8.5–10 ppm) arise from the aldehydes. Aldehyde functionality (9.5–10.0 ppm) was absent from all bio-oils despite the C=O functional groups (1645–1720 cm^−1^) observed by FT-IR. The appearance of such FT-IR bands can also be due to other carbonyl-bearing groups like protonated carboxylic acids, carboxylic acid esters, amides and ketones.

### GC–MS analysis of bio-oil

GC–MS was utilized to further understand the composition of the liquid product and to confirm the findings of the FT-IR and NMR analyses. GC–MS clearly showed that the liquefaction temperature affected the components of bio-oils1. The identification of the main peaks of compounds was performed using the NIST mass spectral database. The HTL experiments resulted in bio-oils which arose from the decomposition of lignin, proteins, flavonoids and lipids along with a range of carbohydrate-derived compounds. The components of bio-oils were identified as mono- and polycyclic aromatic compounds, ketones, aldehydes, esters, alcohols, amides and other nitrogen-containing compounds (including amides and *N*-heterocyclic compounds) and hydrocarbons. A semi-quantitative analysis was performed by calculating the relative percentage of the area of the chromatographic peaks with results shown in Table 3. The main compounds observed from the hydrothermal liquefaction of *A. filiculoides* were methyl pyrazine, 2-methyl 2-cyclopenten-1-one, phenol, 2-methoxy phenol, 3-pyridinol, catechol, l-proline, *N*-butoxy carbonyl- butyl ester, Bis(2-ethylhexyl) phthalate and beta-sitosterol. As the hydrothermal liquefaction temperature varied from 260 to 300 °C, there was production of different compounds as well as different proportions of compounds. The area percentage of phenolic compounds such as phenol (6.1–18.8 area  %) and catechol (10.0–15.7 area  %) increased as the temperature increased from 260 to 280 °C, but then decreased to 11.1 area % for phenol and 11.8 area % for catechol for 300 °C. The phenolics in the bio-oil from *A. filiculoides* were likely produced from the lignin or carbohydrate portion of the biomass [46]. Nitrogenated compounds are formed by decarboxylation, deamination, dehydration, depolymerization and decomposition reactions of proteins [47, 48]. The highest percentage area of 3-pyrindiol (13.3 area %) was observed at the lowest temperature of 260 °C and decreased to 11.9 and 10.1 area  % for 280 and 300 °C, respectively. In addition, highest area about (13.6, 12.4 and 8.7 area %) at 260, 280 and 300 °C was observed. As the composition of the liquid product is so complex, further upgrading, such as denitrogenation and deoxygenation, would be necessary to make the bio-oil suitable for engine fuels.

**Table 3:** GC–MS analysis of HTL-produced bio-oil products from *A. filiculoides*

Compounds identified in bio-oil	Area, %		
	260 °C	280 °C	300 °C
Pyrazine, methyl-	1.4	1.8	2
2-Cyclopenten-1-one, 2-methyl-	1.4	2.6	2
Pyrazine, 2,6-dimethyl-	–	0.8	–
Pyrazine, 2,5-dimethyl-	–	–	1
Phenol	6.1	18.8	11.1
p-Cresol	–	1.4	2.6
2-Cyclopenten-1-one, 2-hydroxy-3-methyl-	1.1	–	–
2-Cyclopenten-1-one, 2,3-dimethyl-	–	1.6	1.3
Phenol, 2-methoxy-	1.1	1.2	1.2
3-Pyridinol	13.3	11.9	10.1
3-Pyridinol, 6-methyl-	1.7	–	2.1
Phenol, 4-ethyl-	–	–	1
Phenol, 4-amino-	–	1.2	
Catechol	10	15.7	11.8
Phenol, 4-ethyl-2-methoxy-	0.8	2.2	–
1,2-Benzenediol, 4-methyl-	–	–	2.8
1,4-Benzenediol, 2-methyl-	–	–	0.6
Naphthalene, 2,6-bis(1,1-dimethylethyl)-	–	–	1.8
Cyclopentane, 1,1,3-trimethyl-3-(2-methyl-2-propenyl)-	–	5	–
Hydantoin, 1-butyl-	3.9	1.9	–
2-Benzimidazolinethione, hexahydro-	1.2	–	–
l-Proline, *N*-butoxycarbonyl-, butyl ester	7.8	7.2	–
l-Proline, *N*-butoxycarbonyl-, heptyl ester	–	–	3.3
l-Norleucine, *N*-allyloxycarbonyl-, octadecyl ester	1.1	–	–
2,5-Piperazinedione, 3-benzyl-6-isopropyl-	1.9	0.6	–
9-Octadecenamide, (Z)-	0.8	1.4	1.5
2,5-Piperazinedione, 3-benzyl-6-isopropyl-	0.9	1.2	–
Cyclo-(l-leucyl-l-phenylalanyl)	3.2	1.9	–
Bicyclo[11.3.0]hexadecane-2,14-dione	2.4	–	–
Bis(2-ethylhexyl) phthalate	13.6	12.4	8.7
Beta-sitosterol	22.8	–	–
Stigmastan-3,5-diene	–	–	8.5

The composition of molecules obtained in the bio-oil from HTL was compared to the spectrum of molecules identified in the pyrolysis-generated bio-oil [41]. Unlike HTL, pyrolysis is the thermochemical decomposition of dry organic matter with a moisture content below 10 % mass fraction in the absence of oxygen, at atmospheric pressure and higher temperatures (350–550 °C) [49]. Both thermochemical technologies showed a great potential for converting the whole Azolla biomass into bio-oils which have higher energy densities than the initial biomass feedstock. Bio-oils from both thermochemical reactions have a very complex composition as a result of depolymerization and decomposition of biomass monomers by cleavage, dehydration, decarboxylation and deamination [49]. Protein-derived compounds were represented by phenols, alkyl phenols, pyrrolidinone, indole and nitrile as a result of decomposition of the amino acids tyrosine and phenylalanine found in Azolla [50]. Lipid-derived products included a range of long-chain saturated alkanes and fatty acids sized from C12 to C21: dodecane, tridecane, tetradecane, pentadecane, hexadecane, heptadecane, octadecane, nonadecane, eicosane and heneicosane. A similar mixture of long-chain lipid-derived alkanes pyrolysis bio-liquid products was found in algae [51–56]. The main difference was that the pyrolysis of *A. filiculoides* showed accumulation of phytol, 3,7,11,15-tetramethyl-2-hexadecen-1-ol, acyclic diterpene alcohol, as the product of the degradation of chlorophyll was the most abundant pyrolysis product.

### Analysis of bio-residue products

**Fig. 7:**
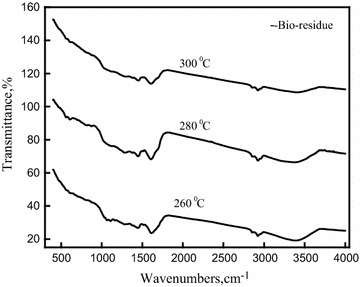
FT-IR of *A. filiculoides* bio-residue obtained at 260, 280 and 300 °C

Figure 7 shows the FT-IR spectra of the Azolla feed and the bio-residues. The broad bands at 3200–3405 cm^−1^ are assigned to the stretching vibrations of hydrogen-bonded O–H groups and N–H groups and indicate the presence of polysaccharides, carbohydrates and proteins present in the Azolla feed. Initial Azolla feeds have strong stretching vibration peaks corresponding to the O–H and N–H groups, but that transmittance decreases in the bio-residue. The peak at 1030 cm^−1^ disappeared in the bio-residue. The peak around 1600–1620 cm^−1^ corresponding to the N–H bending vibration was present in Azolla feed and Azolla bio-residue. The peaks between 2800 and 2930 cm^−1^ in the spectra of the residues became much weaker than those in the Azolla raw feed. The presence of a single peak at 1590 cm^−1^, attributed to the C=C stretching, indicates the formation of aromatic bio-char [57].

The X-ray Powder Diffraction (XRD) spectra of *A.filiculoides* feed and bio-residues at different temperatures showed no significant differences in the powder X-ray diffractogram (Additional file 8: Figure S3). The biomass feed and residue obtained at various temperatures showed typical amorphous nature indicating the structure of carbon in the residue is highly conjugated aromatic sheets cross-linked in a random manner. Morphological changes were apparent in the scanning electron microscopy (SEM) images of the Azolla and bio-residue (Additional file 9: Figure S4). The images of the bio-residue obtained by hydrothermal liquefaction of the three Azolla bio-chars showed that the residue was spongy in nature, and there was no ordered porous structure.

### Hydrogen production from acid-treated Azolla biomass by *Enterobacter cloacae* DT-1

Under heat-H_2_SO_4_ pre-treatment, *A. filiculoides* biomass was hydrolyzed to different soluble reduced sugars mainly consisting of glucose (1.18 g/L), xylose (1.39 g/L) and arabinose (0.063 g/L). The acid-treated pre-hydrolysate used as feedstock for hydrogen production by *Enterobacter cloacae* (*E. cloacae*) in batch mode under decreased partial pressure pH2. pH2 significantly affects the hydrogen yield efficiency of fermentative hydrogen production by anaerobic bacteria since the hydrogen production pathways are sensitive to hydrogen concentration leading to end product inhibition. It has been reported that high hydrogen partial pressure results in the production of more reduced products like ethanol and lactate instead of acetate, butyrate and H_2._ The batch fermenter was arranged to exert pressure on the head space of the fermenter which allowed the biogas to pass out of the head space immediately after its generation, thereby reducing the total partial pressure and thus the hydrogen partial pressure of the batch fermenter.

Fermentation with *E. cloacae* DT-1 led to the production of 53 mmol/L hydrogen (Table 4). During fermentative hydrogen production, the final pH of the fermentation broth dropped from 7.5 to 5.42, which can be attributed to the production of short chain organic fatty acids [58, 59]. The total volatile fatty acid production was 1150 mg/L, largely composed of acetic acid (986 mg/L) and butyric acid (161 mg/L), with B/A ratio of 0.165. Hence the DT-1 strain followed the mixed acid pathway and effectively utilized the C5 sugars (xylose, arabinose and glucose) from the acid-treated prehydrolysate. Hydrogen yield efficiency was 2.43 mol of H_2_/mol of reducing sugar. As the maximum theoretical hydrogen yield efficiency from the dark fermentation route is 4 mol of H_2_/mol of glucose, approximately 60 % of the maximum possible hydrogen yield was obtained from the acid-treated biomass prehydrolysate. To the best of our knowledge, this is the first report for hydrogen production from *A. filiculoides* biomass sugars.

**Table 4:** Batch fermentative hydrogen production by *E. cloacae* DT-1 from *Azolla filiculoides* biomass

Biomass pre-treatment	Hydrogen production (mmol/L)	Final pH	VFA		Total VFA (g/L)	B/A ratio	Yield efficiency, (mol of H_2_/mol of substrate)
			Acetic acid (g/L)	Butyric acid (g/L)			
Acid based pre-treatment	53	5.4	0.986	0.161	1.147	0.16	2.43
Enzymatic hydrolysis	34.82	5.6	0.99	0.164	1.154	0.165	2.04

*VFA* volatile fatty acid

*B/A* butyric acid/acetic acid

### Hydrogen production from enzymatically treated Azolla biomass by *E. cloacae* DT-1

Enzymatic hydrolysis of acid-treated *A. filiculoides* biomass released glucose and xylose at a concentration of 1.49 and 0.181 g/L, respectively. Around 34.8 mmol/L of volumetric hydrogen production (under decreased pH2) was obtained from the enzymatically hydrolyzed biomass sugars. The final pH of the fermentation broth dropped to 5 during hydrogen production which was accompanied by the production of acetic acid (990 mg/L) and butyric acid (164 mg/L). Total VFA concentration was 1156 mg/L and the B/A ratio was 0.165 (Table 4). These results show that the mixed acid fermentation pathway was followed. The hydrogen yield efficiency was 2.04 mol of H_2_/mol of reducing sugar; therefore, it was lower than that obtained from the acid-treated prehydrolysate. Overall, hydrogen yield efficiency of the DT-1 strain from *Azolla filiculoides* biomass was 2.2 ± 2 mol/mol of reducing sugar. The efficiency of hydrogen production from Azolla is similar to that obtained for terrestrial lignocellulosic feedstock which covers the range of 044–2.76 mol H_2_/mol sugars (Additional file 1: Table S1, Additional file 10: Table S6).

The carbon-to-nitrogen (C/N) ratio of biomass is important for efficient anaerobic digestion since microorganisms require significantly more C than N (C:N of 25:1) for optimal hydrogen production. For this reason, the application of microalgae for hydrogen production is limited because they are rich in proteins and have a low C/N ratio (av. of 4) [60]. Co-fermentation with macro-algae (such as *Laminaria digitata*), which are rich in carbohydrates with a high (C/N) ratio, improves the performance of hydrogen production [60]. *A. filiculoides* contains 41 % total carbohydrates and 20 % proteins, as a result proximal analysis of biomass showed a C/N of 15 (Additional file 6: Table S4) which is close to being an efficient feedstock for bio-hydrogen production.

### Ethanol production from *Azolla filiculoides* and *A. pinnata*

Chemical analysis of *A. filiculoides* and *A. pinnata* biomasses is shown in Additional file 3: Table S2. *A. filiculoides* showed a high concentration of biomolecules which can be used as feedstock for production of bio-ethanol: starch (6.05 %), cellulose (21.8 %) and hemicellulose (13.5 %). It has 10.3 % of lignin, which is higher than in duckweed and other aquatic plants but lower than in the main lignocellulosic bioenergy crops (Additional file 1: Table S1). Chemical analysis of *A. pinnata* showed a lower concentration of bio-ethanol-producing molecules: starch (4.7 %), cellulose (12.8 %) and hemicellulose (10.1 %) and higher lignin content (up to 13.2 %).

The starch component of *A. filiculoides* and *A. pinnata* was enzymatically hydrolyzed by α-amylase and α-amyloglucosidase, and the cellulose/hemicellulose components were hydrolyzed by cellulase and cellobiase. The amounts of released glucose are shown in Table 5. As expected, the combination of four enzymes hydrolysing *A. filiculoides* biomass produced a higher amount of glucose than for *A. pinnata* (up to 65.9 and 29.5 g/L, respectively). This glucose yield was much higher than after treatment of the *A. filiculoides* biomass with a suspension of live *Aspergillus niger* grown on potato dextrose broth (2.5 g/L of glucose) shown by Pandey et al. [61].

**Table 5:** Glucose release and ethanol yields from the *Azolla filiculoides* and *A. pinnata* biomasses

Biomass/saccharification	Released glucose, g/L			Ethanol yield, YE/G, g/g^a^	Ethanol yield, YE/B, g/g^b^
*A. filiculoides*					
Biomass, g dw/L	52.2	121.23	256.12	NA	NA
No treatment	ND	ND	ND	ND	ND
α-amylase + α-amyloglucosidase + cellulase + cellobiase	15.9 ± 4.1	31.5 ± 6.6	65.9 ± 11.8	0.56 ± 0.1	0.089 ± 0.02
*A. pinnata*					
Biomass/saccharification	Released glucose, g/L			Ethanol yield, Y E/G, g/g^c^	Ethanol yield, YE/B, g/g^d^
Biomass, g dw/L	52.8	120.78	250.98	NA	NA
No treatment	ND	ND	ND	ND	ND
α-amylase + α-amyloglucosidase + cellulase + cellobiase	6.5 ± 1.0	13.9 ± 3.1	29.5 ± 8.1	0.28 ± 0.03	0.053 ± 0.01

No significant differences were observed between fermentation with or without additional nutrients

*ND* not detected

*NA* not analysed

^a^From 65.9 g/l of glucose

^b^From 256.12 g dw/l of biomass

^c^From 29.5 g/l of glucose

^d^From 250.9 g dw/l of biomass

After fermentation of reduced sugars with *Saccharomyces cerevisiae* the ethanol yields, Y_E/G_ (ethanol/glucose, g/g) reflected the amount of released glucose in the samples, with yields of 0.56 g/g observed for *A. filiculoides* and 0.28 g/g for *A. pinnata.* The yield of 0.56 g/g is comparable to those reported from the fermentation of duckweed hydrolysates and some energy crops [62] (Additional file 1: Table S1). Interestingly, supplementing the fermentation with additional nutrients showed no significant enhancement of the ethanol yield (not shown). This can be explained by the presence of the key nutrients for yeast growth in the biomass of both Azolla species.

The ethanol yield based on *A. filiculoides* biomass (Y_E/B_) was determined as 0.09 g/g which is similar to the yield from *S. cerevisiae* ATCC 24859 grown on the duckweed *L. minor* which contained on average 10 % starch [62]. A higher ethanol yield, 0.19 g/g, was observed for *Lemna aequinoctialis* strain 6000 which has a fast growth rate and the ability to accumulate high levels of starch (up to 39 %) [63]. The potential of another duckweed representative, *Spirodela polyrrhiza,* to increase its starch level under stress conditions up to 45.7 % together with enzymatic hydrolysis with α-amylase, pullulanase and α-amyloglucosidase led to Y_E/B_ of 0.28 g/g [19, 20]. The chemical composition of the cell walls and lower starch accumulation was the likely reason for the lower ethanol yield from *A. pinnata* biomass (Y_E/B_ = 0.05 g/g). Utilization of different yeast strains which can better ferment the spectrum of released sugars, and are more tolerant to the potential inhibitors, will be the next step in the optimization of ethanol yield from Azolla biomass.

### Azolla as universal feedstock for biofuel production

A systematic analysis of terrestrial plants for their potential to be ideal bioenergy crops led to a number of important selection criteria which include: (1) chemical composition and high proportion of biofuel-producing molecules; (2) high growth rates/biomass production; (3) high harvest index/rotation period; (4) ability to grow on marginal lands and lack of competition with agricultural crops for arable lands; (5) high freshwater use efficiency and low growing cost; (6) low harvest cost; and (7) production of high value co-products [64]. These criteria have triggered an intensive search for domestication of the next generation of energy crops.

Azolla species together with their evolutional cyanobacterial symbiont, *A. azollae,* have attracted attention because of their potential to address all of the criteria mentioned above. Firstly, Azolla‘s biomass contains major types of energy molecules which include cellulose/hemicellulose, starch, and lipids resembling combinations of lignocellulosic, starch- and oil-producing terrestrial bioenergy crops and microalgae/cyanobacteria. Secondly, by doubling their biomass every 5–6 days and high annual productivity, they are one of the fastest growing plants, being second after microalgae for biomass production rates. Similar to microalgae their biomass production is not seasonal. Thirdly, Azolla can be grown efficiently outside of their natural habitat using wastewater as their main source of nutrients. Bioremediation of wastewaters can lead to absorption of up to 2.6 and 0.434 t/ha-year of total N and P, respectively [24, 25]. Azolla can also grow efficiently in N-depleted media assimilating up to 0.4 t N/ha-year nitrogen from the atmosphere through symbiosis with *A. azollae*. Fourthly, they are easy to harvest. And finally, Azolla species have been used for decades as nitrogen bio-fertilizers and livestock/fish feed (due to their high protein and carbohydrate levels). The lack of need to use synthetic nitrogen-based fertilizers for growing Azolla makes a substantial positive impact on the reduction of nitrous oxide (N_2_O) generation, the impact of which on warming the atmosphere is almost 300 times that of carbon dioxide [65].

### Thermochemical technologies

Azolla biomass was used as feedstock for production of bio-oil, bio-char and biogas using two thermochemical technologies, pyrolysis [24] and HTL (this work). Given the high annual productivity of Azolla, this leads to the theoretical production of pyrolysis- and HTL-based bio-oils valued as up to 13.2 and 20.2 t/ha-year, respectively. Both bio-oils contain a range of petrochemicals which can be directly used as glycerin-free components of biodiesel or can be upgraded to biofuels using some well-established technologies [24]. Both thermochemical technologies can also produce bio-solids, such as bio-char and ash (up to 48 t/ha-year for both), which can be used to improve soil quality by increasing their nutrient- and moisture-holding capacity, support microbial communities and promote the root activity [66]. Ash from Azolla contains alkali elements (Li, Na, K, Mg, Ca) as well as microelements such as Fe, Mn and Cu making it a good candidate for use as fertilizer [24, 67].

### Biodiesel production

Growing on wastewaters, the annual theoretical yield of crude lipids from *A. filiculoides* can be estimated as 1.68 t/ha-year. This amount can be increased to 8 t/ha-year if the plant will grow under natural conditions with productivity around 100 t dw/ha-year. This oil productivity is significantly higher than from soybean (0.44 t/ha-year), sunflower (0.78 t/ha-year), rapeseed (1.17 t/ha-year) and oil palm (6.0 t/ha-year), but lower than the theoretical yield from microalgae (up to 73 t/ha-year, for *Nannochloropsis* sp.) [28, 29, 68]. As Azolla/*A. azollae* contain a range of C16:0, C18:2 and C18:3 fatty acids, their conversion to methyl esters means that the resultant biodiesel meets the crucial requirements of fuel density, cetane number and iodine value for biodiesel set by the EN14214 standard [28].

### Ethanol production

The theoretical ethanol production from *A. filiculoides* is 9.3 t/ha-year (11.7 × 10^3^ L/ha/year, based on specific volume of ethanol = 0.789 g/mL) which is lower than ethanol production from sugarcane (25 × 10^3^ L/ha-year), close to that from corn stover (13.31 × 10^3^ L/ha-year), but higher than from miscanthus (2.3 × 10^3^ L/ha-year), willow (0.3 × 10^3^ L/ha-year) and poplar (1.3 × 10^3^ L/ha-year) (Additional file 1: Table S1).

### Bio-hydrogen production

The high biomass production yield, chemical composition (C/N ratio) and high bio-hydrogen yield of 2.2 mol/mol substrate make Azolla a competitive feedstock for hydrogen production compared with other bioenergy crops (Additional file 1: Table S1, Additional file 10: Table S6).

## Conclusions

The chemical composition of Azolla’s biomass contains a unique combination of bioenergy molecules found in lignocellulosic, starch- and oil-producing terrestrial bioenergy crops, microalgal and cyanobacterial species. Ability to grow on wastewaters and high growth and productivity rates makes Azolla species a most attractive feedstock for low cost, low energy demanding, near zero maintenance system for production of a wide spectrum of biofuels.

## Methods

### Growing Azolla

*Azolla filiculoides* and *Azolla pinnata* were from RMIT University’s collection of aquatic plants. The plants were collected, rinsed in deionized water and any unwanted debris removed. Experiments were carried out in plastic containers (500 mL) filled with 100 and 50 % of the selenium-supplemented synthetic wastewater (SeSW) (Additional file 4: Table S3). The containers were initially seeded with the same amount of fresh *A. filiculoides,* sufficient to cover the entire water surface with a single layer of fronds. The containers were placed in a 23 °C growth chamber with 16 h photoperiod and a photosynthetic photon flux density of 50 µmol/m^2^/s. The solution in each container was mixed every day. Three replicates were included for each treatment. Destructive sampling was conducted to evaluate the Se, ammonia, nitrate and phosphate contents every day over 5 days. Solution samples were analysed for ammonia cations, nitrate and phosphate anions and pH. Concentrations of cations and anions were measured using the ion chromatography system Dionex ICS-1100 (Thermo Scientific, USA). The dry weights were determined immediately after sampling by drying samples at 80 °C overnight. Growth was monitored by from fresh weight using the following equation: $\mu = {{\left( {\ln N_{{{\text{t}}n}} - \ln N_{{{\text{t}}0}} } \right)} \mathord{\left/ {\vphantom {{\left( {\ln N_{{{\text{t}}n}} - \ln N_{{{\text{t}}0}} } \right)} {\left( {t_{n} - t_{0} } \right)}}} \right. \kern-0pt} {\left( {t_{n} - t_{0} } \right)}}$, where *N*_t*n*_ was the fresh weight and *N*_t0_ was the fresh weight at day 0. The chemical composition of Azolla biomass was analysed by near infrared reflectance spectroscopy (NIRS). Starch was analysed by starch (HK) assay kit (Sigma-Aldrich, USA).

### Selenium extraction and measurements

SeSW samples were collected at the beginning and at the end of the experimental period, acidified with concentrated HNO_3_ to pH 2 and kept at 4 °C. Plants from each treatment were rinsed with Milli-Q water, blotted on filter paper and dried at 70 °C overnight. Dried samples were then ground using a mortar and pestle and aliquots (100 mg) were weighed into glass tubes and digested with HNO_3_ (68.5 %):HClO_4_ (70 %) mixture (1 mL, 10:1, v/v) in a dry heating block at 100 °C for 30 min [69]. After cooling to room temperature, samples were filtered (Whatman number 42, 11 cm) and diluted to 10 mL with Milli-Q water. Plant extracts and wastewater were analysed for total selenium concentration by inductively coupled plasma mass spectrometry (ICP-MS) (Agilent Technologies, Model 4500 series 300).

### Shrimp toxicity test

Adult freshwater glass shrimp, *Paratya australiensis,* from RMIT’s collection were used. The shrimps were acclimatized over 2 weeks before commencing the toxicity tests. They were maintained in 20-L glass tanks containing dechlorinated filtered water at 24 °C, with a 16:8 h light:dark photoperiod and a light intensity of 400 to 600 lx. The shrimps were fed daily with algae wafers and trout pellets. However, they were not fed for 24 h before the commencement of the tests. The toxicity tests were performed under static conditions in 500-mL glass beakers over 96 h. The LC_50_ was calculated for each treatment using a Probit analysis. All the statistical tests were analysed using a ToxRat 3Software (ToxRat Solutions GmbH).

### Ethanol production

#### Enzymatic treatment of Azolla

The samples of *A. filiculoides* and *A. pinnata* which had been washed in MilliQ water was dried in a hot air oven at 70 °C overnight and then ground. For de-lignification, the sample (5 g) was mixed with 25 mM NaOAc (pH 5.5) and autoclaved at 121 °C, 15 lbs, for 20 min. Enzymatic saccharification was performed according to [62]. In brief, 500 µL of a-amylase (Sigma-Aldrich, USA) and 50 µL α-amyloglucosidase (Sigma-Aldrich, USA) were added per gram of dry biomass and incubated at 37 °C with shaking at 250 rpm for 5 h. For the second step, the pH was adjusted to 4.8, and 200 µL of cellulase (Sigma-Aldrich, USA) and 70 µL of glucosidase (Cellobiase) from *Aspergillus niger* (Sigma-Aldrich, USA) per gram of dry biomass were added. The suspension was shaken at 50 °C for 24 h. Glucose released was determined with a glucose assay kit (Sigma-Aldrich, USA).

#### Ethanol fermentation

*Saccharomyces cerevisiae* was grown from a slope culture by inoculation into 200 mL of Difco™ Yeast and Mould (YM) broth 0.3 % (w/v) yeast extract, 0.3 % (w/v) malt extract, 0.5 % (w/v) peptone and 1 % (w/v) dextrose. The yeast was grown in this medium for 3 days at 25 °C. Before inoculation of the hydrolysate, the yeast was centrifuged (3000 rpm, 3 min) and then resuspended in growth media. Ethanol fermentations were carried out by *S. cerevisiae* (1.0 × 10^7^ cells/mL) added to 500 µL of each hydrolysate with and without additional nutrients (0.5 g/L yeast extract and 0.5 g/L peptone) and fermented at 28 °C and 150 rpm for 48 h. Then samples were transferred to screw-cap tubes and were heated at 100 °C for 5 min to terminate the fermentation. Samples were centrifuged, and the supernatants were assessed for ethanol by gas chromatography.

### Bio-hydrogen production

#### Microorganism, media and growth condition

*Enterobacter cloacae* DT-1 (Gene Bank accession number: JX885522) isolated previously was employed [58]. This strain was routinely maintained anaerobically in BSH medium. BSH medium was composed of (g/L): peptone; 2, yeast extract; 1, NaCl_2_; 2, K_2_HPO_4_; 0.230, KH_2_PO_4_; 4.035, 4 mL (4×) of trace solution and 4 mL (4×) of vitamin solution. Trace element solution was composed of (g/L) MnO_4_.7H_2_O; 0.01, ZnSO_4_.7H_2_O; 0.05, H_3_BO_3_; 0.01, N(CH_2_COOH)_3_; 4.5, CaCl_2_.2H_2_0; 0.01, Na_2_MoO_4_; 0.01, CoCl_2_.6H_2_O; 0.01, MgCl_2_.6H_2_O; 00.2, FeCl_3_; 0.1, CuCl_2_.6H_2_O; 0.05. Vitamin solution was composed of (g/L); riboflavin; 0.025, citric acid; 0.02, folic acid; 0.01, and para-amino benzoic acid, 0.01. The pH of the BSH medium was adjusted to 7.5 and incubation temperature was set at the optimum of 37 °C.

#### Acid pre-treatment of *Azolla filiculoides* biomass

Ten grams of dried *A. filiculoides* biomass sample was hydrolysed in 1 % sulphuric acid by autoclaving for 60 min at 120 °C. The hydrolysed biomass was centrifuged at 10,000 rpm for 10 min, and the supernatant (designated as acid-treated prehydrolysate) was separated. The pre-treated solid biomass was processed for enzymatic treatment to obtain the hydrolyzed sugars. The acid-treated prehydrolysate of the *A. filiculoides* biomass sample was analysed for reduced sugar concentration and employed further to use as feedstock for bio-hydrogen production by *E. cloacae* DT-1.

#### Enzymatic saccharification of *Azolla filiculoides* biomass

The pre-treated *A. filiculoides* biomass pellet was processed for enzymatic hydrolysis for its conversion to reducing sugars. The pre-treated *A. filiculoides* biomass sample was acidified with RO water; the pH was reduced to 5 and enzymatic treatment was conducted at 50 °C for 24 h by adding the enzyme cellulase. The hydrolyzed sample was analysed for the concentration of sugar before use as feedstock for dark fermentative bio-hydrogen production by *E. cloacae* DT-1.

#### Batch dark fermentation experiments

Laboratory-scale batch fermentative hydrogen production studies were conducted in 2000 mL serum bottles (batch reactors) containing 160 mL of anaerobically prepared BSH medium [59, 70] supplemented separately with acid-treated prehydrolysate (50 % v/v, 11 g/L of reducing sugars) and enzymatically hydrolyzed sugars (33 % v/v, 4.8 g/L reducing sugar) as feedstock. The initial pH of the media was maintained at 7.5, and 10 % (v/v) freshly grown DT-1 culture was used as inoculum. The bottles were incubated at 37 °C for 72 h under static conditions. Biogas generated during the fermentation process was collected under decreased partial pressure of H_2_ (by reducing the total pressure of biogas in the head space of the fermenter). This entailed using a water displacement system involving an inverted water-filled bottle to exert pressure on the head space of the fermenter which allowed displacement of the biogas immediately after its generation within the batch fermenter. Volumetric biogas production was monitored by measuring the displaced water collected in a graduated inverted water displacement system containing saline solution at ambient temperature. All experiments were performed in duplicate. Qualitative detection of hydrogen was done by gas chromatography.

#### Analytical methods

The composition of the biogas generated in the head space during the dark fermentation process was analysed by gas chromatography (7890A, Agilent Technologies, USA) by following the protocols of Subudhi et al. [58]. High-Performance Liquid Chromatography (HPLC, Agilent 1100 series, USA) using a Sugar-PAK.1 column (Water Research, USA) was used for the detection of ethanol. Water was used as the mobile phase at a flow rate of 0.6 mL/min. All the analyses were performed in duplicate. Sugar concentrations were measured by the DNS method.

### Hydrothermal liquefaction

Hydrothermal liquefaction experiments were conducted in a 100-mL high-pressure autoclave (Parr reactor) made of hastelloy at different reaction conditions of temperature and residence time. In a typical hydrothermal liquefaction experiment, the reactor was loaded with *A. filiculoides* and water as a solvent (1:6 by weight). The reactor was purged five times with nitrogen to remove the air. Reactants were agitated with a stirrer (~200 rpm). The temperature was then raised to the desired value and maintained at that level for 15 min. The pressure during the process was autogenous, with maximum pressure in the range of 60–83 bar under the different reaction conditions. After the reaction, the procedure for separation of bio-oil1, bio-residue and bio-oil2 was as given in [40]. The experiments were repeated several times and the deviation of the liquid yields was within 1 %. The equations used to calculate the yield of various fractions were as follows:

$${\text{Conversion}} \,(\% ) = \frac{W1 - W2}{W1} \times 100$$

$${\text{Bio-oil yield}} \, \left( {{\text{wt}}\% } \right) = \frac{{W {\text{ether soluble}}}}{W1} \times 100$$

$${\text{Bio-oil}} 2 {\text{yield }} \,\left( {{\text{wt\%}}} \right) = \frac{{W {\text{acetone soluble}}}}{W1} \times 100$$

$${\text{Solid residue yield}} \, \left( {{\text{wt}}\% } \right) = \frac{{W {\text{solid}}}}{W1} \times 100$$

$${\text{Gas yield}}\, \left( {{\text{wt}}\% } \right) = \frac{{W \left( {{\text{vessel}} + {\text{feed}} + {\text{water}} } \right)\,{\text{before}} \,{\text{HTU}} - W \left( {{\text{vessel}} + {\text{feed}} + {\text{water}}} \, \right)\, {\text{after}}\, {\text{HTU}}}}{{{\text{Amount}}\, {\text{of}}\, {\text{feed}}\, {\text{taken}} \, ({\text{g}}) + {\text{amount}}\, {\text{of}}\, {\text{water}}\, {\text{added}}\, ({\text{g}})}} \times \, 100$$

$${\text{Other yield}} \,\left( {{\text{wt}}\% } \right) = 100 - \left( {{\text{bio-oil}} 1 + {\text{bio-oil}} 2 + {\text{solid residue}} + {\text{gas}}} \right)$$

where *W1* is the weight of Azolla feed; *W2* is the weight of bio-residue; *W* ether soluble is the weight of ether-soluble bio-oil (bio-oil1); and *W* acetone soluble is the weight of acetone soluble bio-oil (bio-oil2). All yields were calculated by dry material.

TG-DTG of *A. filiculoides* was carried out on a Shimadzu DTG-60 under N_2_ flow. The elemental analysis was conducted in an Elementar Vario micro cube unit. Moisture content was obtained using an HR-83 Mettler Toledo Halogen Moisture Analyzer. The ^1^H NMR spectra were recorded on a Bruker Avance 500 Plus instrument using CDCl_3_ as a solvent. X-ray diffraction patterns of sample powders were collected by a Bruker D8 Advance X-ray diffractometer fitted with a Lynx eye high-speed strip detector and a Cu Kα radiation source. Diffraction patterns in the 2°–80° region were recorded with a 0.04 step size (step time = 4 s). The FT-IR spectra were recorded on a Nicolet 8700 FTIR spectrometer with the sample powder diluted in KBr. SEM images were obtained with an FEI Quanta 200 F using a tungsten filament doped with lanthanum hexaboride (LaB_6_) as an X-ray source, fitted with an ETD (Everhart–Thornley detector), which preferentially worked as a secondary electron detector.

The samples for SEM were dispersed on an adhesive-coated carbon paper and then were gold coated.

The organic fraction of the bio-oil was analysed using gas chromatography–mass spectrometry (GC/MS, Agilent 7890B). The carrier gas was He, and column flow rate was one mL min^−1^. An HP-1 column (25 m × 0.32 mm × 0.17 µm) was used for the separation. The oven was set at 50 °C for 2 min, followed by a heating rate of 5 °C min^−1^–280 °C at which it was held for 5 min. The injected volume was 0.4 µL in splitless mode.

TOC analysis of feed and bio-char was performed using a Shimadzu TOC-L unit with solid sample module SSM-5000A. The volatile matter was calculated by measuring the weight loss in the sample after placing it in a muffle furnace at 950 °C for 2 min, similar to ASTM D3175. Volatile matter and ash analysis of the feed was carried out using oven dried feedstock.

### Statistical analysis

Most of experiments in this study were conducted in triplicate. All data are expressed as mean ± standard deviation. The experimental data were subjected to the one-way analysis of variance (ANOVA) as implemented in the GraphPad InStat 3 statistics platform. Tukey simultaneous tests were conducted to determine the statistical differences between treatments. To ascertain that the observed variations were statistically significant, the probability (*P*) values were determined. A 95 % confidence level (*P* < 0.05) was applied for all analyses.

## Additional files

**Additional file 1: Table S1.** Productivity, chemical compositions, hydrogen and ethanol yields of terrestrial and aquatic feedstocks.

**Additional file 2: Figure S1.** Images of (A) *A. filiculoides* and (B) *A. pinnata*. (C) Filamentous cyanobacterium, *Anabaena azollae* (Aa) squeezed out off *A. filiculoides* leaves (Afl); (D-F) *A. azollae* filaments within *A. filiculoides* vegetative cells. Scale bars: 1 cm for A, B; 20 µm for C; 300 µM, 200 µM and 100 µM for D, E, F, respectively. Red arrow show locations of *A. azollae* filaments; yellow arrow show heterocysts within *A. azollae* filaments.

**Additional file 3: Table S2.** Chemical compositions of *A. filiculoides* and *A. pinnata*.

**Additional file 4: Table S3.** Chemical composition of SeSW.

**Additional file 5: Figure S2.** Survival rates of *P. australiensis* in untreated and treated by *A. filiculoides* SeSW (A); Image of *P. australiensis* (shrimps): live shripmp (left) and dead (right) (B).

**Additional file 6: Table S4.** Proximate and ultimate analysis of *A. filiculoides* biomass, (% DW).

**Additional file 7: Table S5.** Total organic carbon (TOC) analysis of bio-residue from HTL of *A. filiculoides* (at 260, 280 and 300 °C).

**Additional file 8: Figure S3.** Powder XRD of *A. filiculoides* feed and bio-residue obtained from hydrothermal liquefaction of *A. filiculoides* at 260, 280 and 300 °C.

**Additional file 9: Figure S4.** SEM of *A. filiculoides* (A) and bio-residue obtained from hydrothermal liquefaction at 260 °C (B), 280 °C (C) and 300 °C (D).

**Additional file 10: Table S6.** Hydrogen production from terrestrial feedstock.

## Abbreviations

*A. azollae*: *Anabaena azollae* Strasburger
*A. filiculoides*: *Azolla filiculoides*
*A. pinnata*: *Azolla pinnata*
DTA: differential thermal analysis
DW: dry weight
FTIR: Fourier transform infrared spectroscopy
GC-MS: gas chromatography mass spectrometry
HTL: hydrothermal liquefaction
LC50: the lethal concentration at which 50 % of the population if killed in a given period of time
NMR: nuclear magnetic resonance spectroscopy
SEM: scanning electron microscopy
SeSW: selenium-rich synthetic wastewater
TGA: thermogravimetric analyser
TOC: total organic carbon
