# Supplementary material for: Aquatic plant Azolla as the universal feedstock for biofuel production
Source: Biotechnol Biofuels. 2016 Oct 18;9:221. doi: 10.1186/s13068-016-0628-5 (PMC5069886; doi:10.1186/s13068-016-0628-5)
Supplement: Supplementary file 1 — Additional file 1: Table S1. Productivity, chemical compositions, hydrogen and ethanol yields of terrestrial and aquatic feedstocks. [file 13068_2016_628_MOESM1_ESM.docx]

|  | **Table S1:** Productivity, chemical composition, hydrogen and ethanol yield from terrestrial and aquatic feedstocks | | | | | | | | |  |  |  |  |
| --- | --- | --- | --- | --- | --- | --- | --- | --- | --- | --- | --- | --- | --- |
|  |  |  |  |  |  |  |  |  |  |  |  |  |  |
|  | **Species** | **Productivity, t/ha-year** | **Chemical composition, %** | | | | | | **Bio-fuel production** | | | **References** |  |
|  |  |  | **Cellulose** | **Hemicellulose** | **Sucrose/ Starch** | **Total carbohydrates** | **Lignin** | **Lipids** | **Hydrogen, yield** | **Ethanol yield** | |  |  |
|  |  |  |  |  |  |  |  |  |  | **Y E/G, (g/100g substrate)** | **Y E/B, g/g** |  |  |
|  | **Bioenergy crops/grasses** | | | | | | | | | | | |  |
|  | **Sugarcane (a)** | 70 | 55 | 25 | 16(b) | 96 | 24 |  | 1.73 mol H2/mol total sugar | 29.4 | 0.29 | [1, 2] |  |
|  | **Miscanthus** | 6 | 43 | 29 |  | 72 | 19 |  | 2.9 to 3.4 mol H2 per mol of hexose | 37 | 0.3 | [2, 3] |  |
|  | **Switchgrass** | 17 | 37 | 29 |  | 66 | 19 |  | 4 mol H2/mol glucose; 27.1% v/v | 40 |  | [2, 4] |  |
|  | **Corn stover** | 40 | 37 | 26 | 72(c) | 135 | 19 |  | 10.7–12.9 mmol/ L, hr |  | 0.26 | [5, 6] |  |
|  | **Corn kernels** | 5.5 | 16 | 40 | 76.8(c) | 132.8 | 4 |  |  |  | 0.35 | [6-9] |  |
|  | **Woody plants** | | | | | | | | | | | |  |
|  | **Willow** | 15 | 45 | 13 |  | 58 | 13 |  | 0.45 - 0.49 g ethanol/g glucose |  | 0.49 | [10, 11] |  |
|  | **Poplar** | 15 | 43 | 21 |  | 64 | 19 |  | 0.182 g H2/g organics |  | 0.069 | [12] |  |
|  | **Eucalyptus** | 22i-27j | 38 | 27 |  | 165 | 18 |  | 70.80 mmol H2/g raw material | 35 |  | [13, 14] |  |
|  | **Aquatic plants** | | | | | | | | | | | |  |
|  | **Duckweed** | 33 | 43(d) | 4(d) | 10(d); 39(e); 64(f) | 51.2d | 2.4d | 3.1d | 75 mL H2 per g dry | 48.5 | up to 0.28 | [15-18] |  |
|  | **Azolla spp** | 21(g)- 100(h) | 22 | 13 | 6 | 41 | 10 | 7.8 | 2.2 mol per mol glucose/xylose | 40 | 0.09 | [19-20, this paper] |  |
|  | ***Typha spp.*** | 30 | 38.5 | 37 |  | 75.5 | 12.8 |  |  |  |  | [21, 22] |  |
|  | ***Juncus*** | 34 |  |  |  | 0 |  |  |  |  |  | [22] |  |
|  | ***Phragmities*** | 35 | 49 | 31 | 11 | 91 |  |  |  |  | 0.16 | [22,23] |  |
|  | ***Spartina spp.*** | 22 | 0.35 | 0.27 | 46 | 69 |  | 2.3 | 135.9mL/g spartina |  | 0.04 | [24, 25] |  |
|  | **Microalgae** | | | | | | | | | | | |  |
|  | ***Anthrospira platensis*** | 9.8 | 13.6 | 13.2 |  | 40 |  | 7.23 | 93.6 ml H2/g dw |  |  | [26-29] |  |
|  | ***Schiochytrium*** | 14.8 |  |  |  | 17.3 |  | 25-50 |  | 89.8 wt% |  | [30-32] |  |
|  | ***Chlorella spp.*(i)** | 1.5 | 1.09 |  |  |  |  | 43 | 10.8 mL H2/g | 46 | 0.17 | [33-38] |  |
|  | ***Nannochloropsis spp.*** | 1.7 | 25 |  |  |  |  | 30 | 47 mL H2/g/dry biomass |  |  | [39-41] |  |
|  |  |  |  |  |  |  |  |  |  |  |  |  |  |
|  | (a) including bagasse; (b) sucrose; (c) starch; (d) *Lemna minor;* (e) *Lemna aequinoctialis;* (f) *Spirodela polyrrhiza;* (g) growing in maturation pond effluent; (h) growing under natural conditions; (i) heterotrophic conditions | | | | | | | | | | | | |

**Additional file 1**

**Table S1**

|  | | | | |  | | |  | |  |  |  |  |  |  |  |
| --- | --- | --- | --- | --- | --- | --- | --- | --- | --- | --- | --- | --- | --- | --- | --- | --- |
| **References for Table S1** | | | | |  | | |  | |  |  |  |  |  |  |  |
|  | | | |  |  |  |  |  |  |  |  |  |  |  |  |  |
| 1. Pattra, S., et al., Bio-hydrogen production from the fermentation of sugarcane bagasse hydrolysate by *Clostridium butyricum*. International Journal of Hydrogen Energy, 2008. 33(19): p. 5256-5265.  2. Lee, D., et al., Composition of Herbaceous Biomass Feedstocks. 2007, North Central Sun Grant Center, South Dakota State University: Brookings, SD 57007, USA.  3. de Vrije, T., et al., Efficient hydrogen production from the lignocellulosic energy crop Miscanthus by the extreme thermophilic bacteria *Caldicellulosiruptor saccharolyticus* and *Thermotoga neapolitana*. Biotechnology for Biofuels, 2009. 2(1): p. 1-15.  4. Zhang, R., R.C. Brown, and A.A. Suby, Thermochemical Generation of Hydrogen from Switchgrass. Mechanical Engineering Publications, 2004. Paper 155.  5. McAloon, A., et al., Determining the Cost of Producing Ethanol from Corn Starch and Lignocellulosic Feedstocks. 2000, National Renewable Energy Laboratory: Colorado  6. Pioneer Hi-Bred Australia, P.L. Growth Potential Corn Growers Workshop. 2012 [cited 2016 16/06/2016]; Available from: <http://www.pioneer.com/CMRoot/International/Australia_Intl/Publications/Corn_Workshop_Book.pdf>.  7. Abbas, C.A., et al., Process for hydrogen gas production from carbohydrate feedstocks. 2011, Patent US7998455 B2 .  8. Voca, N., et al., Progress in ethanol production from corn kernel by applying cooking pre-treatment. Bioresource Technology, 2009. 100(10): p. 2712-2718.  9. Kim, Y., et al., Composition of corn dry-grind ethanol by-products: DDGS, wet cake, and thin stillage. Bioresource Technology, 2008. 99(12): p. 5165-5176.  10. Bakker, R.R., et al., Biofuel production from acid-impregnated willow and switchgrass, in 2nd World Conference on Biomass for Energy, Industry and Climate Protection, J.W. van Groenestijn and J.H.O. Hazewinkel, Editors. 2004: Rome, Italy.  11. Eklund, R., M. Galbe, and G. Zacchi, The influence of SO2 and H2SO4 impregnation of willow prior to steam pretreatment. Bioresource Technology, 1995. 52(3): p. 225-229.  12. Wang, Z.J., et al., Ethanol production from poplar wood through enzymatic saccharification and fermentation by dilute acid and SPORL pretreatments. Fuel, 2012. 95: p. 606-614.  13. Ferrari, M.D., et al., Ethanol production from eucalyptus wood hemicellulose hydrolysate by Pichia stipitis. Biotechnology and Bioengineering, 1992. 40(7): p. 753-759.  14. Phuhiran, C., T. Takarada, and S. Chaiklangmuang, Hydrogen-rich gas from catalytic steam gasification of eucalyptus using nickel-loaded Thai brown coal char catalyst. International Journal of Hydrogen Energy, 2014. 39(8): p. 3649-3656.  15. Xu, J., et al., Production of high-starch duckweed and its conversion to bioethanol. Biosystems Engineering, 2011. 110(2): p. 67-72.  16. Xu, J., Deshusses, M., Fermentation of swine wastewater-derived duckweed for biohydrogen production. International journal of hydrogen energy, 2015. 40: 7028-7036.  17. Ge XM, Zhang NN, Phillips GC, Xu JF: Growing *Lemna minor* in agricultural wastewater and converting the duckweed biomass to ethanol. Bioresource Technol 2012, 124:485-488.  18. Yu CJ, Sun CJ, Yu L, Zhu M, Xu H, Zhao JS, Ma YB, Zhou GK: Comparative Analysis of Duckweed Cultivation with Sewage Water and SH Media for Production of Fuel Ethanol. Plos One 2014, 9(12).  19. Costa ML, Santos MC, Carrapico F: Biomass characterization of *Azolla filiculoides* grown in natural ecosystems and wastewater. Hydrobiologia 1999, 415:323-327.  20. Brouwer P, van der Werf A, Schluepmann H, Reichart GJ, Nierop KGJ: Lipid Yield and Composition of *Azolla filiculoides* and the Implications for Biodiesel Production. Bioenerg Res 2016, 9(1):369-377.  21. Sopajarn, A. and C. Sangwichien, Optimization of Enzymatic Saccharification of Alkali Pretreated *Typha angustifolia* for Glucose Production International Journal of Chemical Engineering and Applications, 2015. 6(4): p. 232-236.  22. Kresovich, S., et al., The Utilization of Emergent Aquatic Plants for Biomass Energy Systems Development. 1982, Solar Energy Research Institute: Golden, Colorado 80401.  23. Kobbing, J.F., N. Thevs, and S. Zerbe, The utilisation of reed (*Phragmites australis*): a review. Mires and Peat, 2013. 13.  24. Liang, Y.-g., et al., Thermal decomposition kinetics and characteristics of *Spartina alterniflora* via thermogravimetric analysis. Renewable Energy, 2014. 68: p. 111-117.  25. Chen, J., et al., Utilization of an invasive species (*Spartina alternifolia*) in the molded pulp industry, in 23rd Asian-Pacific Weed Science Society Conference. 2011: The Sebel Cairns.  26. Castro, G.F.P.d.S.d., et al., Biomass production by *Arthrospira platensis* under different culture conditions. Food Science and Technology (Campinas), 2015. 35: p. 18-24.  27. Cheng, J., et al., Combination of dark- and photo-fermentation to improve hydrogen production from *Arthrospira platensis* wet biomass with ammonium removal by zeolite. International Journal of Hydrogen Energy, 2012. 37(18): p. 13330-13337.  28. El-Mashad, H.M., Biomethane and ethanol production potential of *Spirulina platensis* algae and enzymatically saccharified switchgrass. Biochemical Engineering Journal, 2015. 93: p. 119-127.  29. Aikawa, S., et al., Direct conversion of Spirulina to ethanol without pretreatment or enzymatic hydrolysis processes. Energy & Environmental Science, 2013. 6(6): p. 1844-1849.  30. Kim, J.K., B.-H. Um, and T.H. Kim, Bioethanol production from micro-algae, *Schizocytrium* sp., using hydrothermal treatment and biological conversion. Korean Journal of Chemical Engineering, 2012. 29(2): p. 209-214.  31. Oilgae. Oilgae.com – Oil & Biodiesel from Algae. 2016 16/06/2016]; Available from: <http://www.oilgae.com/algae/oil/yield/yield.html>.  32. Ethier, S., et al., Continuous culture of the microalgae *Schizochytrium limacinum* on biodiesel-derived crude glycerol for producing docosahexaenoic acid. Bioresource Technology, 2011. 102(1): p. 88-93.  33. Chia, M., A.T. Lombardi, and M.d.g. Melao, Growth and biochemical composition of *Chlorella vulgaris* in different growth media. Anais da Academia Brasileira de Ciências, 2013. 85: p. 1427-1438.  34. Liang, F., et al., Growth Rate and Biomass Productivity of Chlorella as Affected by Culture Depth and Cell Density in an Open Circular Photobioreactor. Journal of Microbiology and Biotechnology, 2013. 23(4): p. 539-544.  35. Moncada, J., et al., Production of Bioethanol Using *Chlorella vulgaris* Cake: A Technoeconomic and Environmental Assessment in the Colombian Context. Industrial & Engineering Chemistry Research, 2013. 52(47): p. 16786-16794.  36. Kumar, V.B., et al., Evaluation of the Potential of *Chlorella vulgaris* for Bioethanol Production. Energy & Fuels, 2016. 30(4): p. 3161-3166.  37. Lakaniemi, A.-M., et al., Biogenic hydrogen and methane production from *Chlorella vulgaris* and *Dunaliella tertiolectabiomass*. Biotechnology for Biofuels, 2011. 4(1): p. 1-12.  38. Cheng, Y., et al., Biodiesel production from Jerusalem artichoke (*Helianthus Tuberosus L*.) tuber by heterotrophic microalgae *Chlorella protothecoides.* Journal of Chemical Technology & Biotechnology, 2009. 84(5): p. 777-781.  39. Sukarni, et al., Potential and properties of marine microalgae *Nannochloropsis oculata* as biomass fuel feedstock. International Journal of Energy and Environmental Engineering, 2014. 5(4): p. 279-290.  40. Nobre, B.P., et al., A biorefinery from *Nannochloropsis* sp. microalga – Extraction of oils and pigments. Production of biohydrogen from the leftover biomass. Bioresource Technology, 2013. 135: p. 128-136.  41. Chiu, S.-Y., et al., Lipid accumulation and CO2 utilization of *Nannochloropsis oculata* in response to CO2 aeration. Bioresource Technology, 2009. 100(2): p. 833-838. | | | | | |  | | |  |  |  |  |  |  |  |  |
|  | | | | | |  | | |  |  |  |  |  |  |  |  |
|  |  |  |  | | | |  |  |  |  |  |  |  |  |  |  |
