# Supplementary figures and images for: Aquatic plant Azolla as the universal feedstock for biofuel production
Source: Biotechnol Biofuels. 2016 Oct 18;9:221. doi: 10.1186/s13068-016-0628-5 (PMC5069886; doi:10.1186/s13068-016-0628-5)

**Additional file 2**

**Figure S1**


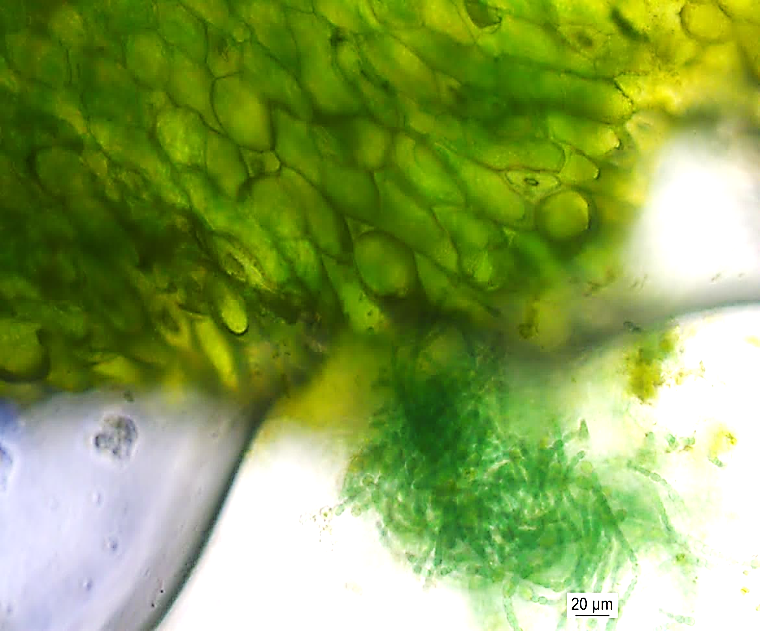

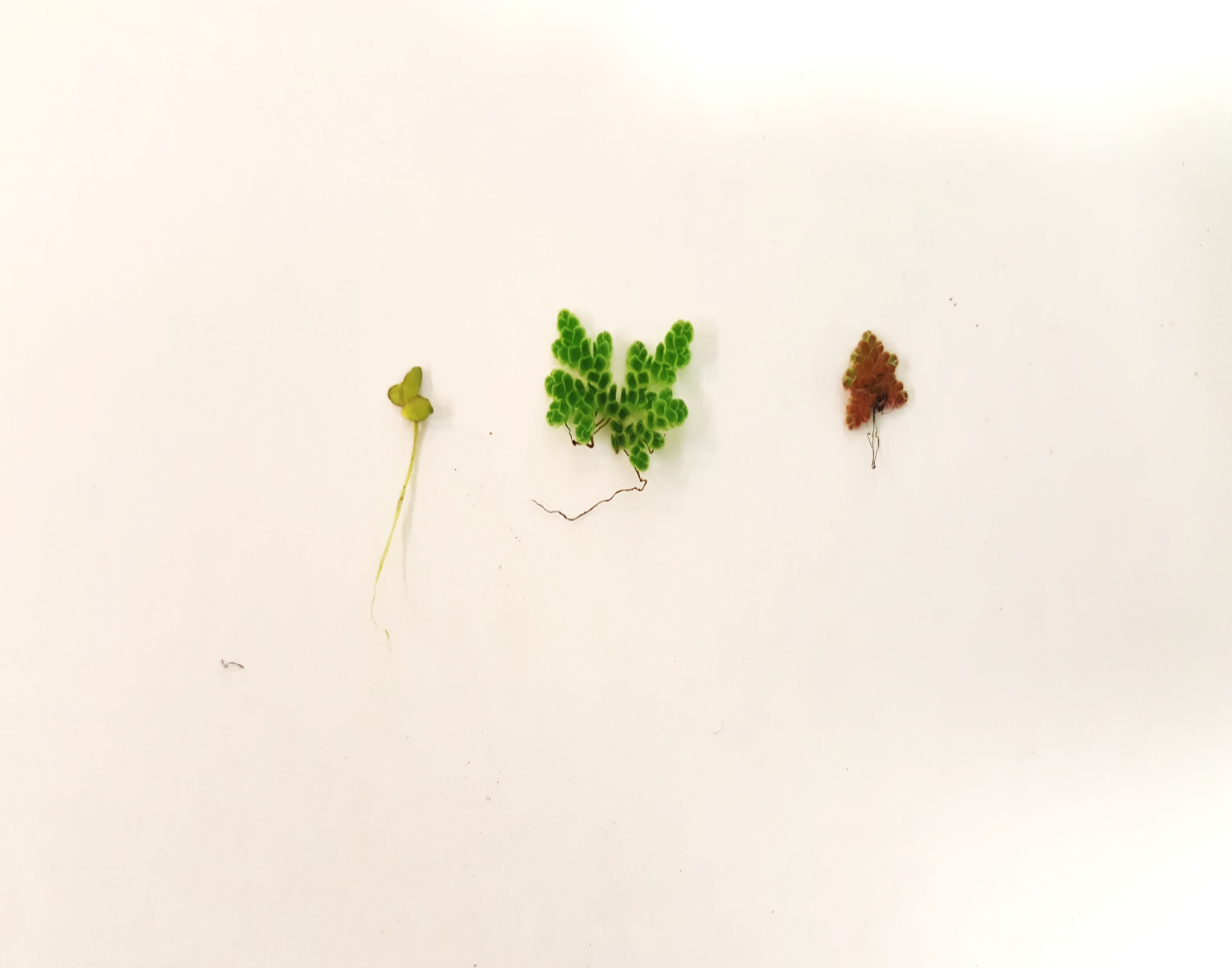

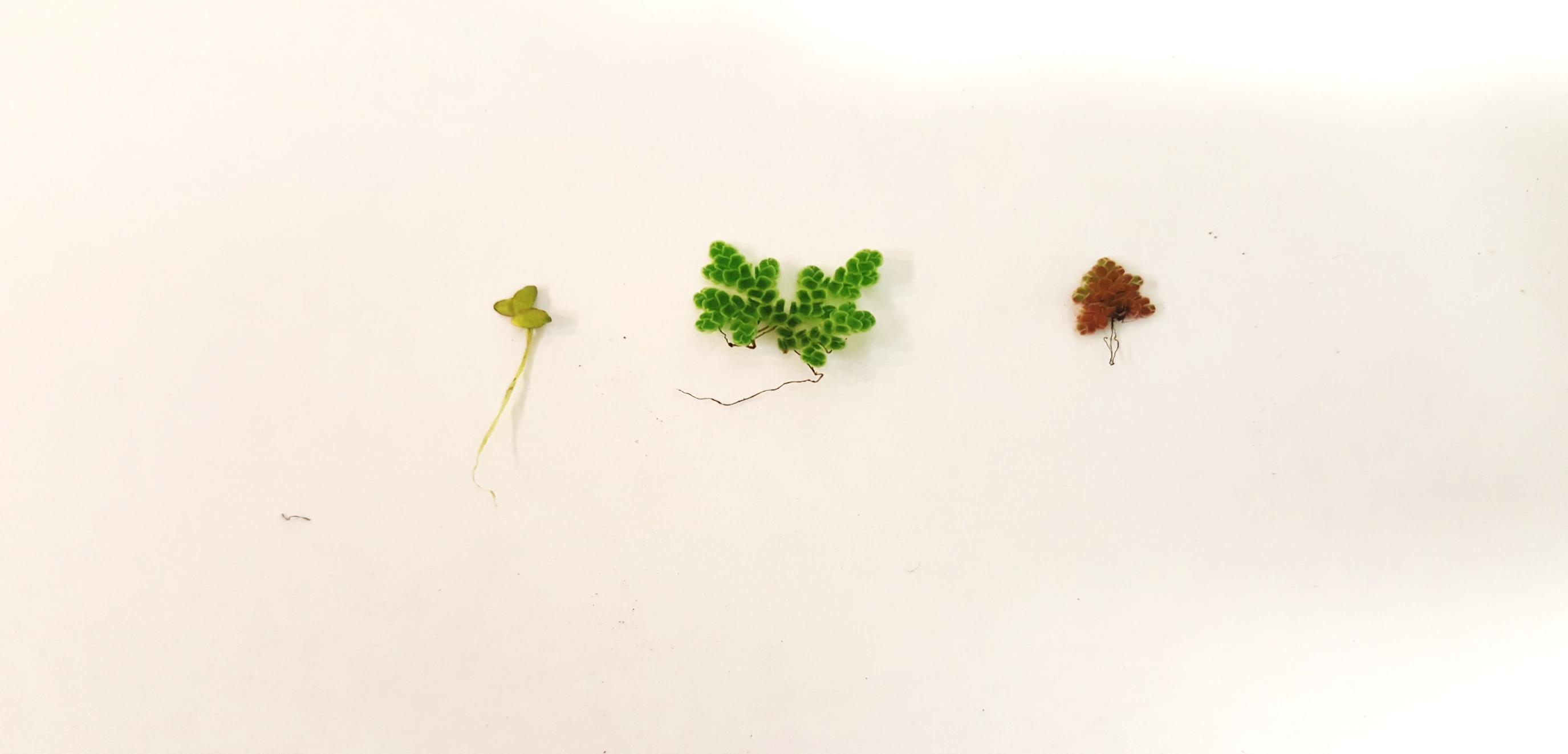

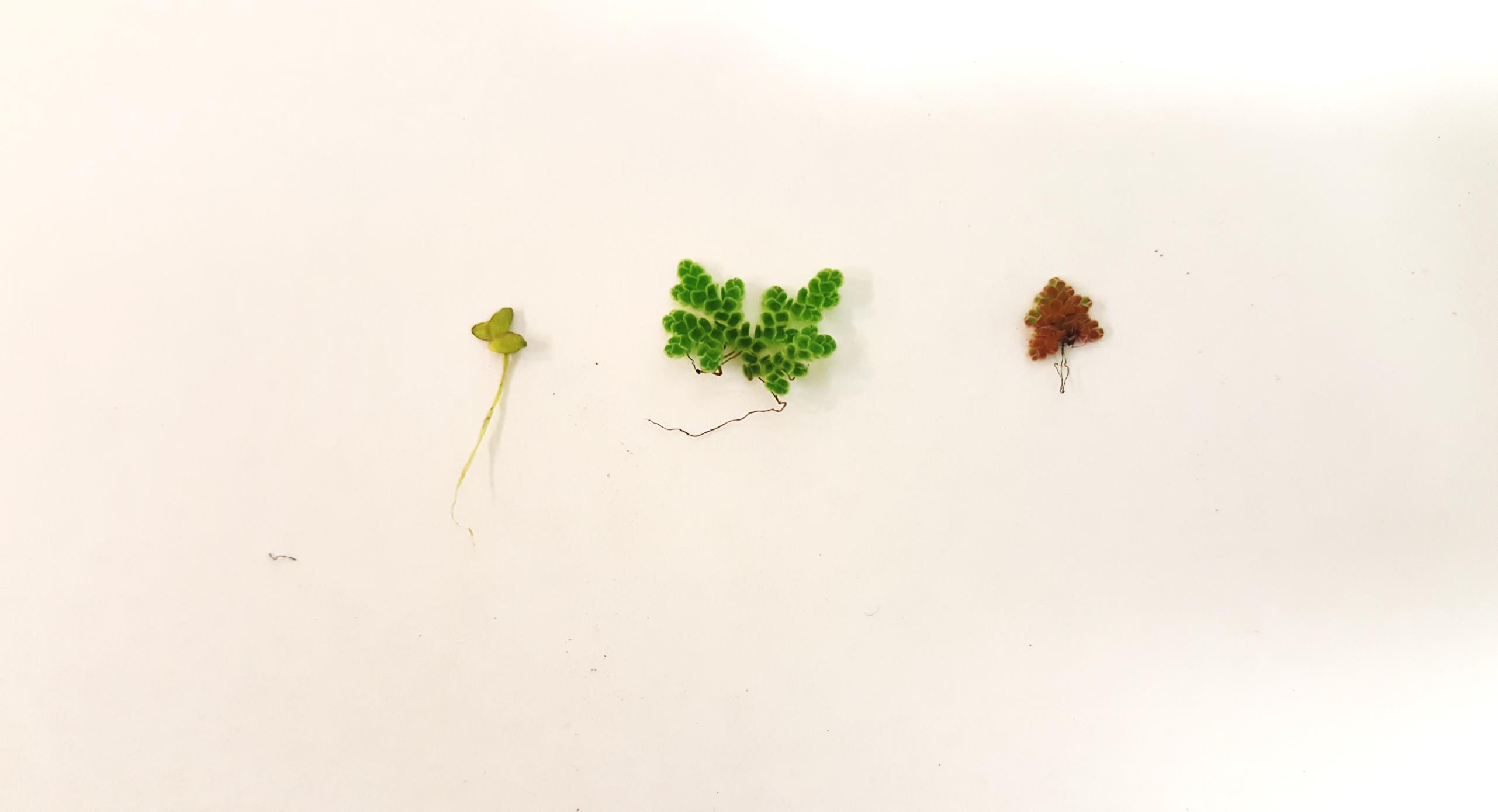


A

B

C

**Afl**


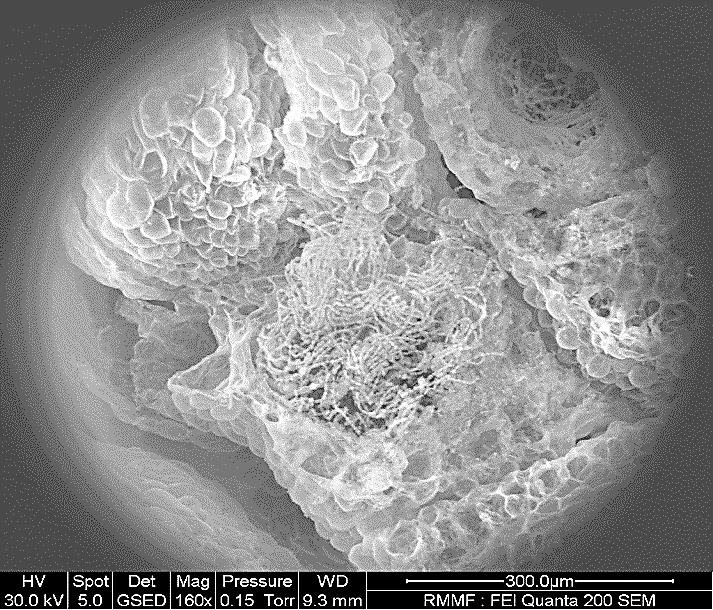

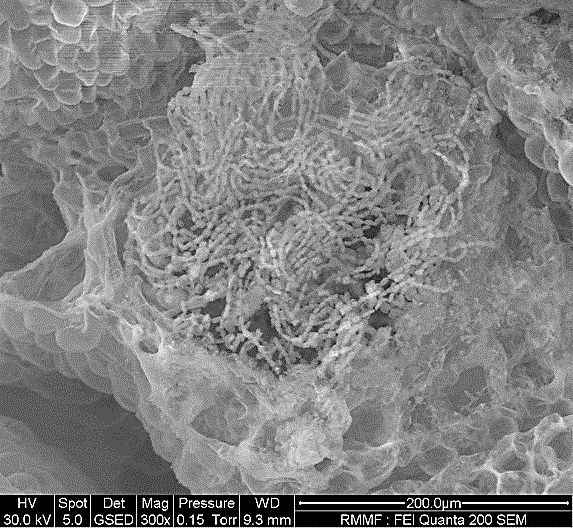

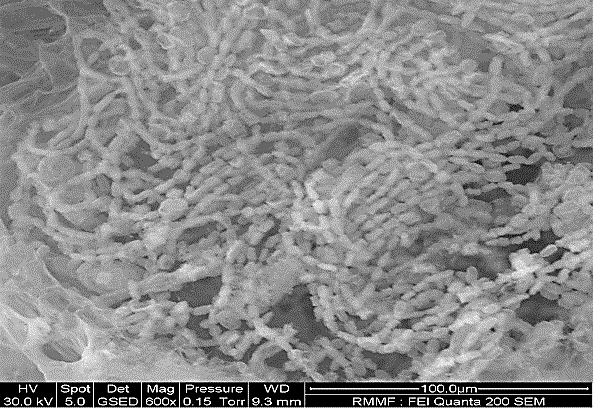


D

E

F

**Afl**

**Afl**

Supplement: Supplementary file 2 — Additional file 2: Figure S1. Images of (A) A. filiculoides and (B) A. pinnata. (C) Filamentous cyanobacterium, Anabaena azollae (Aa) squeezed out off A. filiculoides leaves (Afl); (D-F) A. azollae filaments within A. filiculoides vegetative cells. Scale bars: 1 cm for A, B; 20 µm for C; 300 µM, 200 µM and 100 µM for D, E, F, respectively. Red arrow show locations of A. azollae filaments; yellow arrow show heterocysts within A. azollae filaments. [file 13068_2016_628_MOESM2_ESM.docx]

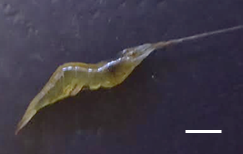


**Additional file 5**

**Figure S2**

Untreated SeSW

SeSW treated by *A. filiculoides*


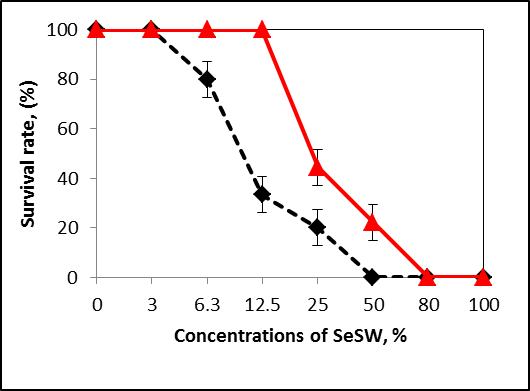


LC50


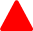


A


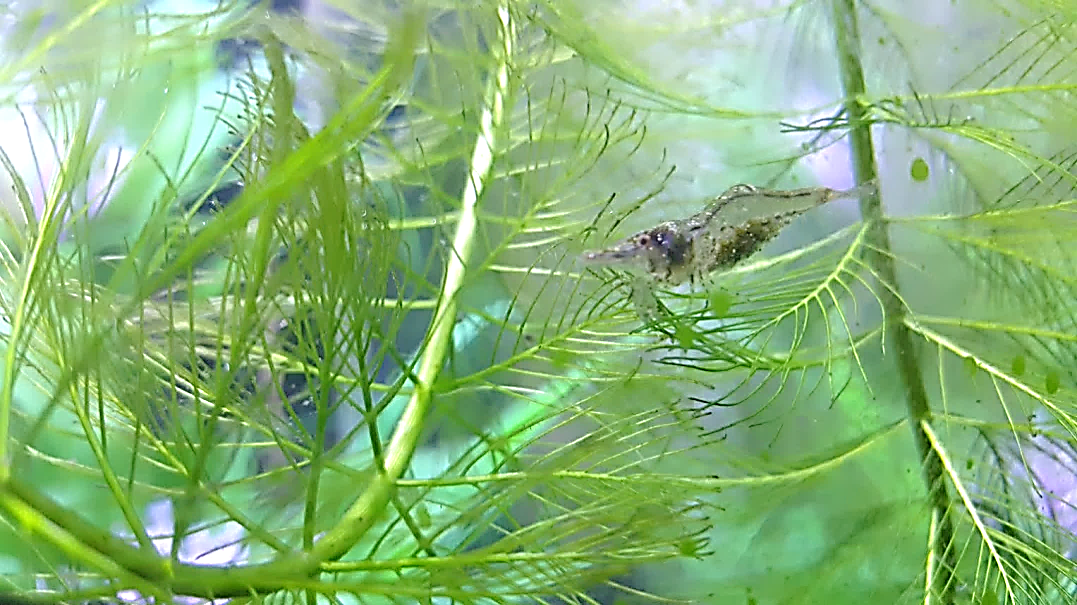


B

Supplement: Supplementary file 5 — Additional file 5: Figure S2. Survival rates of P. australiensis in untreated and treated by A. filiculoides SeSW (A); Image of P. australiensis (shrimps): live shripmp (left) and dead (right) (B). [file 13068_2016_628_MOESM5_ESM.docx]

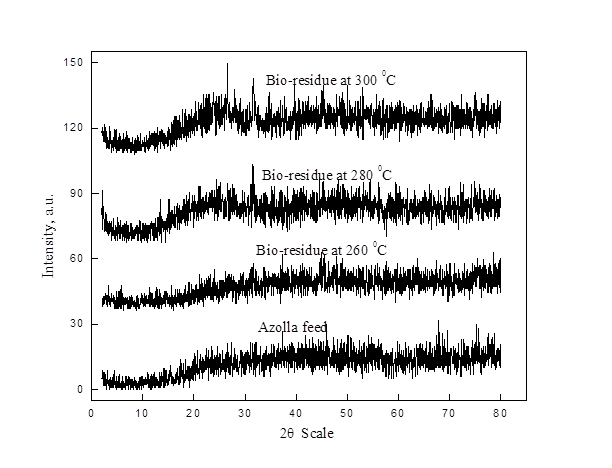


**Additional file 8**

**Figure S3**

Supplement: Supplementary file 8 — Additional file 8: Figure S3. Powder XRD of A. filiculoides feed and bio-residue obtained from hydrothermal liquefaction of A. filiculoides at 260, 280 and 300 °C. [file 13068_2016_628_MOESM8_ESM.docx]

**Additional file 9**

**Figure S4**


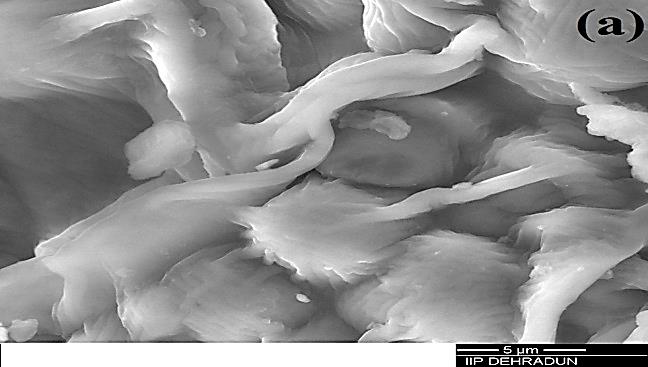

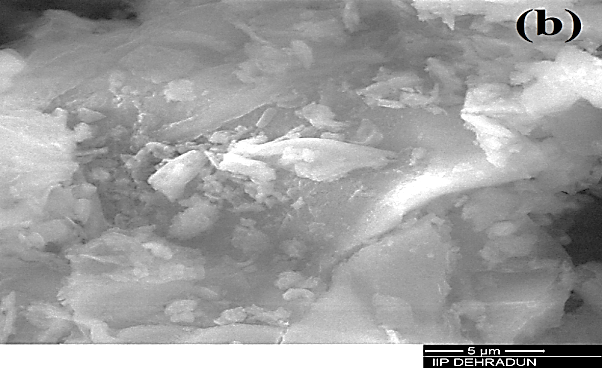

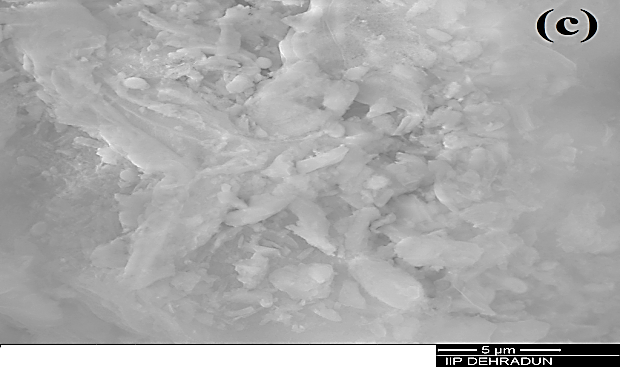

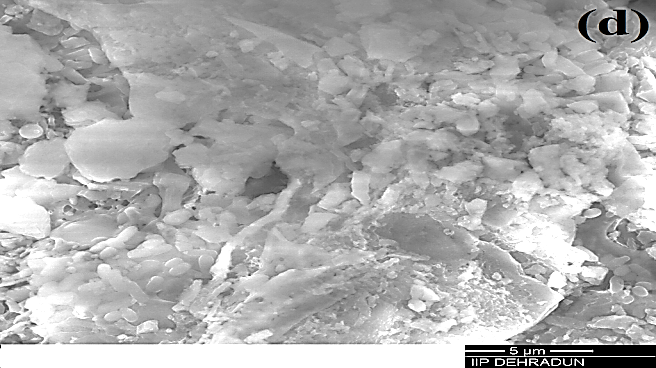


A

B

C

D

Supplement: Supplementary file 9 — Additional file 9: Figure S4. SEM of A. filiculoides (A) and bio-residue obtained from hydrothermal liquefaction at 260 °C (B), 280 °C (C) and 300 °C (D). [file 13068_2016_628_MOESM9_ESM.docx]
