# Supplementary material for: Aquatic plant Azolla as the universal feedstock for biofuel production
Source: Biotechnol Biofuels. 2016 Oct 18;9:221. doi: 10.1186/s13068-016-0628-5 (PMC5069886; doi:10.1186/s13068-016-0628-5)
Supplement: Supplementary file 3 — Additional file 3: Table S2. Chemical compositions of A. filiculoides and A. pinnata. [file 13068_2016_628_MOESM3_ESM.docx]

| **Table S2.** Chemical composition of *A. filiculoides* and *A. pinnata* | | | |
| --- | --- | --- | --- |
|  |  |  |  |
| **Component** | ***A. filiculoides*** | ***A. pinnata*** |  |
| **Ash, %** | 12.4 | 16.8 |  |
| **Protein, %** | 21.80 | 25.1 |  |
| **Organic matter, %** | 83.10 | 89.1 |  |
| **Dry matter, %** | 5.12 | 8.1 |  |
| **Crude fat, %** | 7.52 | 4.1 |  |
| **Cellulose, %** | 21.80 | 12.8 |  |
| **Hemicellulose, %** | 13.50 | 10.1 |  |
| **Starch, %** | 6.05 | 4.7 |  |
| **Lignin, %** | 10.30 | 13.2 |  |
|  |  |  |  |

**Additional file 3**

**Table S2**
