# Supplementary material for: Aquatic plant Azolla as the universal feedstock for biofuel production
Source: Biotechnol Biofuels. 2016 Oct 18;9:221. doi: 10.1186/s13068-016-0628-5 (PMC5069886; doi:10.1186/s13068-016-0628-5)
Supplement: Supplementary file 4 — Additional file 4: Table S3. Chemical composition of SeSW. [file 13068_2016_628_MOESM4_ESM.docx]

| **Table S3.** Chemical composition of SeSW | |
| --- | --- |
|  |  |
| **Ingredients** | **Concentration per L** |
| KH_2_PO_4_ | 0.585 g |
| K_2_HPO_4_ | 0.218 g |
| Na_2_HPO_4_. 7H2O | 0.503 g |
| NaNO_3_ | 0.015 g |
| NH_4_Cl | 0.055 g |
| CaCl_2_ | 0.0275 g |
| MgSO_4_.7H2O | 0.0225 g |
| Se0_2_ | 800 µg |

**Additional file 4**

**Table S3**
