# Supplementary material for: Aquatic plant Azolla as the universal feedstock for biofuel production
Source: Biotechnol Biofuels. 2016 Oct 18;9:221. doi: 10.1186/s13068-016-0628-5 (PMC5069886; doi:10.1186/s13068-016-0628-5)
Supplement: Supplementary file 6 — Additional file 6: Table S4. Proximate and ultimate analysis of A. filiculoides biomass, (% DW). [file 13068_2016_628_MOESM6_ESM.docx]

| **Table S4.** Proximate and ultimate analysis of *A. filiculoides* biomass, (% DW) | | | |
| --- | --- | --- | --- |
|  |  |  |  |
| **Proximate analysis** | | **Ultimate analysis** | |
| C | 46.2 | Moisture | 11.1 |
| H | 7.4 | Ash content | 7.4 |
| O | 43.2 | Volatile matter | 88.4 |
| N | 3 | Fixed carbon | 4.5 |
| S | 0.2 |  |  |

**Additional file 6**

**Table S4**
