# Supplementary material for: Aquatic plant Azolla as the universal feedstock for biofuel production
Source: Biotechnol Biofuels. 2016 Oct 18;9:221. doi: 10.1186/s13068-016-0628-5 (PMC5069886; doi:10.1186/s13068-016-0628-5)
Supplement: Supplementary file 7 — Additional file 7: Table S5. Total organic carbon (TOC) analysis of bio-residue from HTL of A. filiculoides (at 260, 280 and 300 °C). [file 13068_2016_628_MOESM7_ESM.docx]

| **Table S5:** Total organic carbon (TOC) analysis of bio-residue from | | | | |  |
| --- | --- | --- | --- | --- | --- |
| HTL of *A. filiculoides*  (at 260 °C, 280 °C and 300 °C). | | | |  |  |
|  |  |  |  |  |  |
| **Sample name** | **Char (gm) A** | **TOC of char (%), B** | **TOC char (gm), C=(AxB)/100** | **Char TOC % , E=C/Dx100** | **Conversion, % (TOC based)= 100-E** |
| **AZ-260** | 4.01 | 60.1 | 2.41 | 86.07 | 13.93 |
| **AZ-280** | 2.28 | 67.7 | 1.54 | 55 | 45 |
| **AZ-300** | 2.03 | 70.4 | 1.43 | 51.07 | 48.93 |
|  |  |  |  |  |  |
| Weight of feed taken=6 gm, TOC of feed= 46.9 %. TOC of the feed (g) (D) =(6x46.9)/100=2.8 gm | | | | | |

**Additional file 7**

**Table S5**
