# Supplementary material for: Aquatic plant Azolla as the universal feedstock for biofuel production
Source: Biotechnol Biofuels. 2016 Oct 18;9:221. doi: 10.1186/s13068-016-0628-5 (PMC5069886; doi:10.1186/s13068-016-0628-5)
Supplement: Supplementary file 10 — Additional file 10: Table S6. Hydrogen production from terrestrial feedstock. [file 13068_2016_628_MOESM10_ESM.docx]

| **Table S6.** Hydrogen production from terrestrial feedstock | | |  |
| --- | --- | --- | --- |
|  |  |  |  |
| **Substrate** | **Inoculum** | **Yield** | **References** |
| Cellulose | *Clostridium cellulolyticum* | 1.7 mol H2/mol hexose consumed | [[1](#_ENREF_1)] |
| Cellulose | *Clostridium termitidis* | 0.62 mol H2/mol hexose | [[2](#_ENREF_2)] |
| Wheat starch | Anaerobic digester sludge | 1.3 mol H2/mol hexose consumed | [[3](#_ENREF_3)] |
| Sugarcane bagasse | *Clostridium butyricum* | 1.73 mol H2/mol total sugar | [[4](#_ENREF_4)] |
| Sugarcane bagasse | Elephant dung | 0.84 mol H2/mol total sugar | [[5](#_ENREF_5)] |
| Wheat straw | Enrichment culture | 1.59 mole H2/mole of hexose | [[6](#_ENREF_6)] |
| Wheat straw | Enrichment culture | 2.56 mole H2/mole of hexose | [[6](#_ENREF_6)] |
| Corn stover | Digested sludge | 2.84 mole H2/mole of hexose | [[7](#_ENREF_7)] |
| Corn stalks | *Clostridium acetobutylicum* | 82 L/kg | [[8](#_ENREF_8)] |
| Corn stalks | *Clostridium butyricum* | 68 L/kg | [[9](#_ENREF_9)] |
| Cassava pulp | *Clostridium butyricum, Enterobacter aerogenes* | 2.76 mole H2/mole of hexose | [[10](#_ENREF_10)] |
| Wheat starch | Anaerobic sludge | 2.84 mole H2/mole of hexose | [[11](#_ENREF_11)] |
| Wheat straw | Compost | 68 L/kg | [[12](#_ENREF_12)] |
| Rice straw | Sludge | 0.44 mole H2/mole of hexose | [[13](#_ENREF_13)] |
| Bagasse | *Clostridium pasteurianum* | 0.96 mole H2/mole of hexose | [[14](#_ENREF_14)] |
| Corn stalk waste | Enrichment culture | 122 L/kg | [[15](#_ENREF_15)] |
| Beet-pulp | Anaerobic sludge | 0.79 mole H2/mole of hexose | [[16](#_ENREF_16)] |
| Rice straw | Sewage treatment plant | 0.95 mole H2/mole of hexose | [[17](#_ENREF_17)] |

**Additional file 10**

**Table S6**

**References for Table S6**

1. Ren Z, Ward TE, Logan BE, Regan JM: Characterization of the cellulolytic and hydrogen-producing activities of six mesophilic Clostridium species. J Appl Microbiol 2007, 103(6):2258-2266.

2. Ramachandran U, Wrana N, Cicek N, Sparling R, Levin DB: Hydrogen production and end-product synthesis patterns by *Clostridium termitidis* strain CT1112 in batch fermentation cultures with cellobiose or α-cellulose. Int J Hydrogen Energ 2008, 33(23):7006-7012.

3. Hussy I, Hawkes FR, Dinsdale R, Hawkes DL: Continuous fermentative hydrogen production from a wheat starch co-product by mixed microflora. Biotechnol Bioeng 2003, 84(6):619-626.

4. Pattra S, Sangyoka S, Boonmee M, Reungsang A: Bio-hydrogen production from the fermentation of sugarcane bagasse hydrolysate by *Clostridium butyricum*. Int J Hydrogen Energ 2008, 33(19):5256-5265.

5. Fangkum A, Reungsang A: Biohydrogen production from sugarcane bagasse hydrolysate by elephant dung: Effects of initial pH and substrate concentration. Int J Hydrogen Energ 2011, 36(14):8687-8696.

6. Kongjan P, Angelidaki I: Extreme thermophilic biohydrogen production from wheat straw hydrolysate using mixed culture fermentation: Effect of reactor configuration. Bioresource Technol 2010, 101(20):7789-7796.

7. Datar R, Huang J, Maness P-C, Mohagheghi A, Czernik S, Chornet E: Hydrogen production from the fermentation of corn stover biomass pretreated with a steam-explosion process. Int J Hydrogen Energ 2007, 32(8):932-939.

8. Ren N, Wang A, Gao L, Xin L, Lee D-J, Su A: Bioaugmented hydrogen production from carboxymethyl cellulose and partially delignified corn stalks using isolated cultures. Int J Hydrogen Energ 2008, 33(19):5250-5255.

9. Li D, Chen H: Biological hydrogen production from steam-exploded straw by simultaneous saccharification and fermentation. Int J Hydrogen Energ 2007, 32(12):1742-1748.

10. Phowan P, Reungsang A, Danvirutai P: Bio-hydrogen Production from Cassava Pulp Hydrolysate using Co-culture of *Clostridium butyricum* and *Enterobacter aerogenes*. Biotechnology 2010, 9(3):348-354.

11. Yokoyama H, Moriya N, Ohmori H, Waki M, Ogino A, Tanaka Y: Community analysis of hydrogen-producing extreme thermophilic anaerobic microflora enriched from cow manure with five substrates. Appl Microbiol Biot 2007, 77(1):213-222.

12. Chu C-Y, Wu S-Y, Tsai C-Y, Lin C-Y: Kinetics of cotton cellulose hydrolysis using concentrated acid and fermentative hydrogen production from hydrolysate. Int J Hydrogen Energ 2011, 36(14):8743-8750.

13. Liu C-M, Chu C-Y, Lee W-Y, Li Y-C, Wu S-Y, Chou Y-P: Biohydrogen production evaluation from rice straw hydrolysate by concentrated acid pre-treatment in both batch and continuous systems. Int J Hydrogen Energ 2013, 38(35):15823-15829.

14. Cheng X-Y, Liu C-Z: Fungal pretreatment enhances hydrogen production via thermophilic fermentation of cornstalk. Appl Energ 2012, 91(1):1-6.

15. Guo Y-P, Fan S-Q, Fan Y-T, Pan C-M, Hou H-W: The preparation and application of crude cellulase for cellulose-hydrogen production by anaerobic fermentation. Int J Hydrogen Energ 2010, 35(2):459-468.

16. Ozkan L, Erguder TH, Demirer GN: Effects of pretreatment methods on solubilization of beet-pulp and bio-hydrogen production yield. Int J Hydrogen Energ 2011, 36(1):382-389.

17. Chang ACC, Tu Y-H, Huang M-H, Lay C-H, Lin C-Y: Hydrogen production by the anaerobic fermentation from acid hydrolyzed rice straw hydrolysate. Int J Hydrogen Energ 2011, 36(21):14280-14288.

**Additional file 10**

**Table S6**
